# A novel variant in *SMG9* causes intellectual disability, confirming a role for nonsense-mediated decay components in neurocognitive development

**DOI:** 10.1038/s41431-022-01046-5

**Published:** 2022-01-28

**Authors:** Elisa Rahikkala, Lea Urpa, Bishwa Ghimire, Hande Topa, Mitja I. Kurki, Maryna Koskela, Mikko Airavaara, Eija Hämäläinen, Katri Pylkäs, Jarmo Körkkö, Helena Savolainen, Anu Suoranta, Aida Bertoli-Avella, Arndt Rolfs, Pirkko Mattila, Mark Daly, Aarno Palotie, Olli Pietiläinen, Jukka Moilanen, Outi Kuismin

**Affiliations:** 1 Department of Clinical Genetics, PEDEGO Research Unit and Medical Research Center Oulu, Oulu University Hospital and University of Oulu, Oulu, Finland; 2 Institute of Biomedicine, University of Turku, Turku, Finland; 3 Institute for Molecular Medicine Finland (FIMM), University of Helsinki, Helsinki, Finland; 4 Neuroscience Center, Helsinki Institute of Life Science, University of Helsinki, Helsinki, Finland; 5 Psychiatric & Neurodevelopmental Genetics Unit, Massachusetts General Hospital, Boston, MA USA; 6 The Stanley Center for Psychiatric Research, The Broad Institute of MIT and Harvard, Cambridge, MA USA; 7 Division of Pharmacology and Pharmacotherapy, Faculty of Pharmacy, University of Helsinki, Helsinki, Finland; 8 Cancer and Translational Medicine Research Unit and Biocenter Oulu, University of Oulu, NordLab Oulu, Oulu, Finland; 9 Center for Intellectual Disability Care, Oulu University Hospital, Oulu, Finland; 10 Centogene GmbH, 18055 Rostock, Germany; 11 Medical Faculty, University of Rostock, Rostock, Germany; 12 Analytic and Translational Genetics Unit, Department of Medicine, Massachusetts General Hospital, Boston, MA USA; 13 Department of Neurology, Massachusetts General Hospital, Boston, MA USA

**Keywords:** Gene expression, Genetics of the nervous system, Disease genetics

## Abstract

Biallelic loss-of-function variants in the *SMG9* gene, encoding a regulatory subunit of the mRNA nonsense-mediated decay (NMD) machinery, are reported to cause heart and brain malformation syndrome. Here we report five patients from three unrelated families with intellectual disability (ID) and a novel pathogenic *SMG9* c.551 T > C p.(Val184Ala) homozygous missense variant, identified using exome sequencing. Sanger sequencing confirmed recessive segregation in each family. *SMG9* c.551T > C p.(Val184Ala) is most likely an autozygous variant identical by descent. Characteristic clinical findings in patients were mild to moderate ID, intention tremor, pyramidal signs, dyspraxia, and ocular manifestations. We used RNA sequencing of patients and age- and sex-matched healthy controls to assess the effect of the variant. RNA sequencing revealed that the *SMG9* c.551T > C variant did not affect the splicing or expression level of *SMG9* gene products, and allele-specific expression analysis did not provide evidence that the nonsense mRNA-induced NMD was affected. Differential gene expression analysis identified prevalent upregulation of genes in patients, including the genes *SMOX, OSBP2, GPX3*, and *ZNF155*. These findings suggest that normal SMG9 function may be involved in transcriptional regulation without affecting nonsense mRNA-induced NMD. In conclusion, we demonstrate that the *SMG9* c.551T > C missense variant causes a neurodevelopmental disorder and impacts gene expression. NMD components have roles beyond aberrant mRNA degradation that are crucial for neurocognitive development.

## Introduction

Pathogenic variants in nonsense-mediated mRNA decay (NMD) components in humans have been associated with intellectual disability (ID) and disruption of normal development [1–3]. NMD is a selective RNA turnover mechanism maintaining steady-state RNA levels, degrading both aberrant mRNAs harboring premature translation termination codons (PTCs) and subsets of normal mRNAs, including in neural development [4, 5]. NMD is a complex pathway involving numerous components regulated in a tissue-specific and developmentally controlled manner [6]. This has raised interest in the developmental role of NMD components and whether disrupting the degradation of aberrant transcripts disturbs normal brain development.

X-linked, recessively inherited loss-of-function variants in the core NMD component *UPF3B* (OMIM 300298) result in ID [1], whereas autosomal recessive pathogenic variants in NMD regulators *SMG9* and *SMG8* have been associated with heart and brain malformation syndrome (OMIM 616920) [2, 7]. To date, seven patients from five consanguineous families carrying homozygous pathogenic *SMG9* variants, unique to their pedigree, have been described [2, 8–10]. In the current study, we report and delineate the phenotypic spectrum of a further five Finnish patients from three unrelated families harboring the same novel homozygous *SMG9* c.551T > C missense variant, which is enriched in the Finnish population. The phenotype is associated with mild to moderate ID, mild dysmorphisms, dyspraxia, increased susceptibility to a heart defect, and pyramidal signs. The phenotype is similar, but milder than in the previously reported patients with *SMG9* homozygous loss-of-function variants, suggesting a hypomorphic role of the *SMG9* c.551T > C variant.

The mechanism for how pathogenic *SMG9* variants affect the NMD pathway and lead to heart and brain malformation syndrome is largely unknown. *SMG9* encodes a regulatory cofactor of the SMG1 complex, which is essential in NMD, but it is unclear how the altered function of SMG9 affects the NMD mechanism as a whole [11]. To investigate the impact of this *SMG9* variant on the NMD mechanism and the human transcriptome, we compared RNA sequencing results of our five patients with the homozygous *SMG9* c.551T > C variant with that of age- and sex-matched healthy controls.

## Subjects and methods

### Subjects and study approval

A total of 966 patients with either ID or pervasive and specific developmental disorders (ICD-10 codes F70–79 and F80–89, respectively) of unknown etiology who belonged to the Northern Finland Intellectual Disability cohort were recruited for clinical and molecular genetic studies. A detailed description of the project is provided by Kurki et al. [12]. Two patients who were identified using whole exome sequencing (WES) performed as part of clinical diagnostics at Centogene (Rostock, Germany) were also recruited for the project and included in the detailed phenotypic analysis. All patients were examined by one of the authors (ER).

### DNA sequencing

We used standard methods to extract genomic DNA from the peripheral blood samples of the probands and their participating affected or unaffected healthy relatives. WES of DNA samples from Patients 1–3 and Control 3 was performed at the Broad Institute of MIT and Harvard (Cambridge, MA, USA). WES of DNA samples from Patients 4 and 5 and Control 2 was performed at Centogene (Rostock, Germany). More details of the WES analyses are provided in the supplementary note, including detailed clinical data. We confirmed the presence and segregation of the *SMG9* c.551T > C p.(Val184Ala) variant by PCR, followed by conventional Sanger sequencing of DNA samples from patients and their unaffected parents, siblings, and other close relatives who had consented to participate in the study.

### RNA sequencing and data analysis

We used standard methods to extract RNA from the peripheral blood samples of the five probands and five age- and sex-matched healthy control individuals (Supplementary Table 1). RNA sequencing and analysis were performed at the Institute for Molecular Medicine Finland (FIMM, Helsinki, Finland).

We performed differential gene expression and differential transcript analysis using a standard pipeline created by the FIMM Sequencing Center. Briefly, we normalized raw gene count data and detected differentially expressed genes (DEGs) with the edgeR package in R [13], and performed differential transcript analysis using Ballgown [14] with transcripts reconstructed from Stringtie [15]. We used all 13,822 protein-coding genes from the DEG analysis to perform a ranked list enrichment analysis, using the Gene Ontology (GO), Kyoto Encyclopedia of Genes and Genomes (KEGG) and Reactome pathway databases [16–18] from the Molecular Signatures Database [19]. We tested ranked genes for overrepresented pathways using the FGSEA-multilevel method [20]. Detailed descriptions of the RNA sequencing, RNA data analysis, and enrichment analysis are provided in the supplementary note.

### Allele-specific expression analysis

We performed allele-specific expression analysis on allele counts from the ASEReadCounter tool from the Genome Analysis Toolkit [21]. Using the resulting allele counts, we compared the proportion of reference reads for likely NMD-targeted protein-truncating (i.e. nonsense or stop-gain) variants to the proportion of reference reads for other, non-protein-truncating variants and for likely NMD-escaping protein-truncating variants. Allele-specific expression was calculated in Patients 1–4 and Controls 2–3, individuals for which WES data was available to locate the likely NMD-targeted variants. We accounted for the increased likelihood of allele-specific expression from variants on the same gene by randomly choosing one variant per gene per sample, permuted 1000 times. Detailed descriptions of the definition of likely NMD-targeted and likely NMD-escaping genes and the allele-specific expression analysis are provided in the supplementary note.

## Results

### Clinical delineation of the patients

**Fig. 1:**
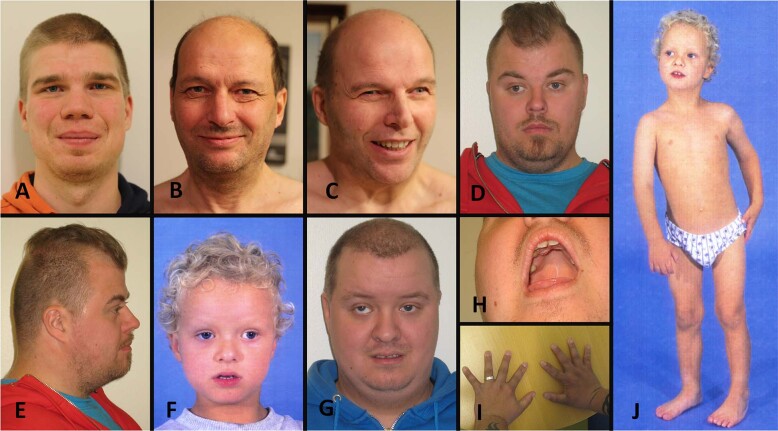
Clinical features of the patients. **A** Facial figure of Patient 1 shows a broad nose and low nasal bridge. **B** Facial figure of Patient 2 shows a low insertion of columella, long nose, and depressed nasal bridge. **C** Facial figure of Patient 3 shows a low insertion of columella, long nose, and depressed nasal bridge. He has alternating exotropia in his eyes. **D**, **E** Facial figures of Patient 4 show a brachycephalic skull, depressed nasal bridge, and exotropia in his right eye. **F**, **G** Facial figures of Patient 5 at the age of 4 and 26 years show facial muscular hypotonia, prominent forehead, depressed and wide nasal bridge, broad nasal tip, low insertion of columella, alternating exotropia in both eyes, and hypertropia in his right eye. **H** The palate of Patient 5 was high and narrow. **I** The hands of Patient 4 show brachydactyly. **J** Patient 5 at the age of 4 years showing his gestalt and planovalgus.

Clinical findings of the five patients with the homozygous *SMG9* c.551T > C p.(Val184Ala) variant are compiled in Table 1 and Fig. 1. All patients were male, aged 24–56 years, and had either mild or moderate ID (*N* = 5/5, 100%). Motor development was typically mildly delayed and two patients were reported to have had muscular hypotonia during childhood (*N* = 2/5, 40%). Language development was markedly delayed in all patients (*N* = 5/5, 100%). First words were delayed in all patients until 2 to 3.5 years, and expressive language increased slowly. The intelligible speech was typically acquired by the age of 6–8 years. Four patients had strabismus (*N* = 4/5, 80%), and three patients had vertical strabismus (*N* = 3/5, 60%). One patient had short stature (*N* = 1/5, 20%), otherwise growth parameters were normal. One patient had a complex congenital heart defect (*N* = 1/5, 20%). All patients had muscular hypertonia and clonic or very brisk reflexes in their lower limbs. In addition, they had intention tremor and slow diadochokinesis (*N* = 5/5, 100%). Four patients were described as ataxic when they were children (*N* = 4/5, 80%). Three patients had planovalgus (*N* = 3/5, 60%). The electroencephalogram (EEG) of three patients showed abnormalities (*N* = 3/5, 60%), but none of the patients were diagnosed with epilepsy.

**Table 1:** Clinical features of patients with a homozygous *SMG9* gene variant c.551T > C p.(Val184Ala).

	Patient 1	Patient 2	Patient 3	Patient 4	Patient 5
**Age**	25 y	56 y	54 y	29 y	26 y
**Sex**	Male	Male	Male	Male	Male
**Birth length**	51 cm (−0.1 SD)	52 cm (+0.5 SD)	NA	50 cm (−0.6 SD)	46 cm (−2.7 SD)
**Birth weight**	3,590 g (−1%)	3,550 g (−8%)	3450 g	3270 g (−4%)	2535 g (−6%)
**Birth OFC**	35.5 cm (+0.2 SD)	NA	NA	35.5 cm (+0.2 SD)	32 cm (−2.4 SD)
**Current height**	183 cm (+0.4 SD)	172 cm (−1.4 SD)	181 cm (0.0 SD)	171 cm (−1.6 SD)	165 cm (−2.6 SD)
**Current weight**	78 kg (+11%)	73 kg (+22%)	73.4 kg (+7%)	85 kg (+44%)	75 kg (+40%)
**Current OFC**	59.7 cm (+1.8 SD)	57 cm (−0.3 SD)	59 cm (+1.0 SD)	54.5 cm (−1.8 SD)	55 cm (−1.7 SD)
**Congenital heart defect**	No	No	No	TGA, VSD, ASD, PDA, hypoplastic RA	No
**Muscular hypotonia**	No	No	No	Yes	Yes
**Tendency to fall as a child**	Yes	Yes	Yes	Yes	Yes
**Independent walking age**	1 y and 3 m	1 y and 8 m	1 y and 3 m	1 y and 6 m	1 y and 7 m
**First words age**	2 y	2 y	3.5 y	2.5 y	3 y
**Language development**	Markedly delayed	Markedly delayed	Markedly delayed	Markedly delayed	Markedly delayed
**AAC methods**	Signs, pictures, gestures used as a child	No	No	Gestures used as a child	Signs, pictures used as a child
**Oral motor dyspraxia**	Yes	Yes	Yes	Yes	Yes
**Severity of ID**	Mild	Borderline mild/moderate	Moderate	Moderate	Moderate
**Word-finding difficulties**	Yes	No	Mild	No	Mild
**Epilepsy**	No	No	No	No	No
**EEG**	N/A	Spike and slow-wave complexes	Bitemporal symmetric spike and slow-wave discharges	N/A	Slow background activity, spike and slow-wave complexes
**Brain MRI**	Mild dilatation of LV, mild lack of WM in the trigonum	N/A	N/A	Normal	Normal
**Tendon reflexes**	Clonic ankle reflexes, brisk patella reflexes	Clonic ankle reflexes, brisk patella reflexes	Clonic ankle reflexes, brisk patella reflexes	Clonic ankle reflexes, brisk patella and brachioradialis reflexes	Clonic ankle reflexes, brisk patella and brachioradialis reflexes
**Intention tremor**	+	+	++	++	+
**Ataxia**	No	Yes, as a child	Yes, as a child	Yes, as a child	Yes, as a child
**Diadochokinesis**	Slow	Slow	Slow	Slow	Slow
**Increased muscle tone in lower limbs**	+	++	++	++	+
**Strabismus**	Alternating esotropia, hypertropia (L), hypotropia (R), operated	No	Alternating exotropia, operated	Exotropia (R), hypertropia (R), no operation	Exotropia (R), hypertropia (R), operated
**High palate**	Yes	No	No	Yes	Yes
**Planovalgus**	Yes	No	No	Yes	Yes
**Infections**	Recurrent otitis media infections before school age	One otitis media infection before school age	One otitis media infection before school age	Recurrent otitis media infections before school age	Recurrent otitis media infections before school age
**Dysmorphism**	Depressed nasal bridge, broad nose	Low insertion of columella, long nose, depressed nasal bridge	Low insertion of columella, long nose, depressed nasal bridge	Brachycephaly, depressed nasal bridge	Prominent forehead, depressed and wide nasal bridge, broad nasal tip, low insertion of columella
**Other**	Mild scoliosis	High BP, hypercholesterolemia	High BP, type 2 DM, benign PH	Brachydactyly	Sacral dimple, brachydactyly

Characteristic features were ID, pyramidal signs, and ocular manifestations. Abbreviations: *y* years, *m* months, - not present, + present, ++ markedly present, *SD* standard deviation from the mean, *NA* not available, *OFC* occipitofrontal circumference, *TGA* transposition of the great arteries, *VSD* ventricular septal defect, *ASD* atrial septal defect, *PDA* patent ductus arteriosus, *RA* right atrium, *ID* intellectual disability, *LV* lateral ventricles, *WM* white matter, *BP* blood pressure, *DM* diabetes mellitus, *PH* prostate hyperplasia, *L* left, *R* right.

Consistent with previously published patients with pathogenic biallelic *SMG9* variants [2, 8–10], the patients had ID, increased peripheral muscle tone, brisk deep tendon reflexes, and dysmorphic facial features including prominent forehead, broad nasal bridge, and high arched palate (Fig. 1). All the previously published patients with pathogenic biallelic *SMG9* variants had a congenital heart defect (*N* = 7/7, 100%) and most of them had brain abnormalities (*N* = 6/7, 86%) [2, 8–10]. Unlike previously published patients with both heart and brain malformations, only one patient (*N* = 1/5, 20%) in this cohort had a congenital heart defect, and one other patient (*N* = 1/5, 20%) had mild dilation of lateral ventricles and mild lack of white matter in the trigonum area. Detailed clinical descriptions of the patients are provided in the supplementary note.

### Genetic results

All five Finnish patients were found to carry a homozygous *SMG9* gene variant: c.551T > C p.(Val184Ala) (NM_019108.4, GRCh38 g.19:43747479 A > G, rs749498958). The variant was identified by WES and confirmed by Sanger sequencing. The variant is present in the Genome Aggregation Database (gnomAD v.2.1.1), with a minor allele frequency of 0.0001627. Due to recent population bottlenecks and subsequent rapid population expansion in isolation [22], the allele frequency is 34 times higher in the Finns (0.001598) than in non-Finnish Europeans (0.00004644), and there are no homozygous individuals in the gnomAD. The expected number of *SMG9* c.551T > C homozygous patients in Finland is (0.001598)^2^*5.5 million is approximately 14 individuals.

**Fig. 2:**
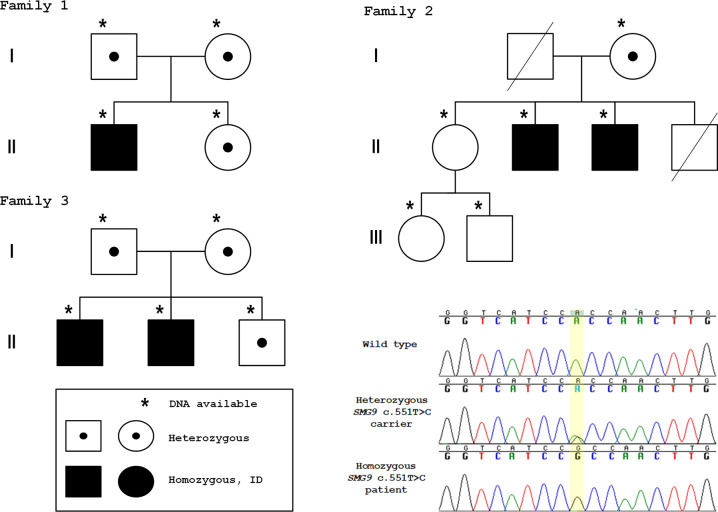
Pedigrees of the families indicating genotypes of the individuals who were available for genetic testing. Sanger sequence traces of the *SMG9* gene variant c.551T > C p.(Val184Ala) showing a healthy control individual (wild type), a healthy heterozygous carrier, and a homozygous patient. Reverse sequences are shown.

The ID segregated in the families in a recessive manner (Fig. 2). To investigate whether the *SMG9* c.551T > C had arisen from a single mutational event, we performed a haplotype analysis using previously published data [23]. The *SMG9* gene variant c.551T > C lies on a shared ancestral haplotype with a median length of 1.7 Mb for shared haplotype segments further suggesting *SMG9* c.551T > C to be a Finnish founder variant.

**Fig. 3:**
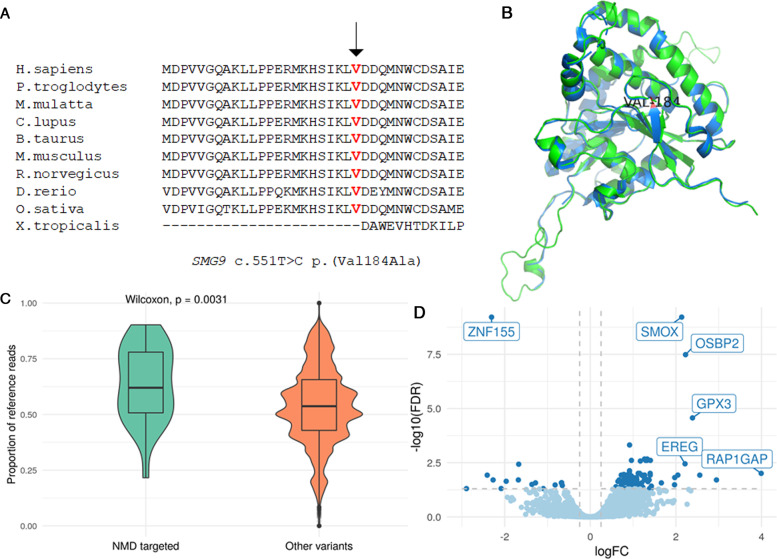
Evidence for the effect of *SMG9* c.551T > C p.(Val184Ala) variant. **A** Multispecies alignment showing the strong conservation of *SMG9* p.Val184 **B** The predicted SMG9-Val184Ala mutated structure (green) superimposed on the wild-type SMG9 structure (blue) showing a predicted structural alteration. **C** Plot comparing the proportion of reference reads from allele-specific expression analysis in likely NMD-targeted variants (*n* = 20) compared to non-protein-truncating variants (*n* = 27,046) in Patients 1–4. The proportion of reference reads was significantly higher in predicted NMD-targeted variants than other variants, suggesting a functional PTC-induced NMD system in patients. **D** Volcano plot showing DEGs between the patients and healthy controls. The overall pattern observed was the increased expression of genes in the patient group compared with the control group. The dark blue dots show statistically significant DEGs (FDR padj < 0.05 and |logFC| > = 0.25). The boxes show the genes that display both large magnitude fold-changes (FC) and high statistical significance (FDR padj).

The *SMG9* c.551T > C variant affects a highly conserved amino acid and a moderately conserved nucleotide (Fig. 3A), and its CADD score is 23.8. In silico, the variant is predicted to be disease causing (Mutation Taster), benign (Polyphen), deleterious (SIFT), and potentially capable of altering of splicing (Human Splicing Finder). We performed an in silico assessment of the SMG9-Val184Ala mutated structure using SWISS-MODEL [24] that predicted a structural alteration compared to the wild-type SMG9 structure (Fig. 3B, Supplementary Fig. 1 and Supplemental video). In the predicted model of mutated SMG9 p.184Ala structure, the magnesium ion is not conserved and there is a structural defect in the G-domain, which is the ATP binding site. This structural change could affect the binding of UPF1 to SMG1-SMG8-SMG9 protein complex, altering the kinase activity of SMG1.

### Classification according to ACMG/AMP Criteria

The *SMG9* c.551T > C allele is significantly enriched in ID patients compared to the general Finnish population (OR = 8.08, 95% CI 3.78–17.29, *p* < 0.001, Supplementary Table 2), providing strong evidence for pathogenicity (PS4). The variant is also at extremely low frequency in the gnomAD (PM2), and co-segregates with the phenotype in multiple affected family members in the known disease-causing gene *SMG9* (PP1), particularly notable considering the very low frequency of the variant. Multiple lines of computational evidence (see above) support a deleterious effect on the gene product (PP3). According to the ACMG/AMP criteria [25], the *SMG9* c.551T > C variant is classified as likely pathogenic (PS4, PM2, PP1, PP3).

### RNA sequencing and allele-specific expression analysis results

We hypothesized that the *SMG9* p.(Val184Ala) missense variant leads to a loss of SMG9 function, which given SMG9′s role in NMD may inhibit PTC-induced mRNA degradation in cells and affect NMD-related transcriptional regulation. To study the biological impact of the *SMG9* variant, we compared the transcriptome of *SMG9* p.(Val184Ala) homozygous patients and healthy controls, who were either *SMG9* p.(Val184Ala) heterozygous or wild type (Supplementary Tables 3, 4 and Supplementary Fig. 2). We first asked whether the *SMG9* c.551T > C would lead to the removal of transcripts harboring the variant. Interestingly, the missense variant did not significantly impact the total mRNA levels or abundances of the different isoforms of the gene (Supplementary Table 5), suggesting that the clinical phenotype in patients was not resulting from loss of SMG9 transcripts.

We then wondered whether the missense variant would result in disrupted splicing of SMG9 gene products. All exons of the *SMG9* gene were found to be expressed in the patient group, and no change in expression levels was detected between the two groups when analyzed on the gene level or transcript level.

To determine the effect of the *SMG9* variant on PTC-induced NMD, we examined allele specific expression (ASE) of genes harboring heterozygous protein-truncating variants that were predicted to be targeted by NMD. We reasoned that for genes where NMD effectively removes the PTC-containing transcripts, the majority of the detected mRNA originates from the transcript containing the reference allele. As recently shown by others [26] this results in deviation from the expected equal biallelic expression for these genes from each parental chromosome. In contrast, if PTC-induced NMD was attenuated by the *SMG9* p.(Val184Ala) missense variant in patients, then both alleles would be present in equal proportions, giving a reference read ratio of approximately 0.5. We identified 28 likely NMD-targeted variants in four patients and two controls. Surprisingly, we saw that the likely NMD-targeted variants in patients had a significantly higher proportion of reference reads than non-NMD targeted variants (*p* = 3.1e−3), indicating a functioning PTC-induced NMD mechanism (Fig. 3C). We did not see a significantly higher proportion of reference reads in NMD-targeted variants in the controls, likely due to the small number of predicted NMD-targeted variants in two controls for which WES was available (*p* = 0.27, Supplementary Fig. 3). Importantly, the protein-truncating variants that were predicted to escape the NMD mechanism did not have significantly higher proportion of reference reads than other variants (*p* = 0.021, Supplementary Fig. 4).

We next asked whether the *SMG9* p.(Val184Ala) missense variant impacts the function of cells by analyzing differential gene expression of 13,822 protein-coding genes in the patient and control data set (Supplementary Table 6). The DGE analysis revealed that the expression of 112 genes was statistically significantly different (false discovery rate adjusted p-value, FDR padj < 0.05) between the patients and the healthy controls (Supplementary Table 7). This analysis revealed genes whose expression is affected by dysfunctional SMG9, which are also candidates possibly contributing to the disease pathogenesis. The overall pattern observed was an increased expression of genes in the patient group compared to the control group, including ~ 6.5 times more upregulated (97) than downregulated (15) genes (p-value = 7.12e−16) (Supplementary Table 7), suggesting that dysfunctional SMG9 either affects NMD-regulated gene expression outside of the known canonical mechanisms or through a non-NMD regulatory mechanism.

The most significant upregulated genes were *spermine oxidase (SMOX)* (logFC = 2.1, FDR padj = 6.1E−10), *oxysterol binding protein 2 (OSBP2)* (logFC = 2.2, FDR padj = 3.3E−08), and *glutathione peroxidase 3 (GPX3)* (logFC = 2.4, FDR padj = 2.7E 05) (Fig. 3D and Table 2). The most significant downregulated gene, with an FDR padj of 6.1E–10 and a logFC of −2.3, was *zinc finger protein 155 (ZNF155)* (Fig. 3D and Table 2). DEGs highly expressed in the brain, eye, and/or heart and with a logFC of ≥ 2.0 or ≤ −2.0 and an FDR padj ≤ 0.05 are shown in Table 2. In the patients’ samples, there was increased expression of genes involved in metabolism, protection from oxidative damage, neuronal differentiation, Notch signaling, apoptosis, mitochondrial metabolism, and microtubule assembly. On the other hand, patients’ samples showed decreased expression of genes involved in transcriptional regulation, axonal growth in the central nervous system, RNA binding, neuromodulation, and microtubule structure.

**Table 2:** Differentially expressed genes that are known to be highly expressed in the brain, eye, and/or heart and had a log fold-change (logFC) of ≥ 2.0 or ≤ −2.0 and false discovery rate (FDR) of ≤0.05.

*Gene*	Gene name	logFC	FDR	Gene function	NMD features
*SMOX*	Spermine oxidase	2.1	6.1E−10	SMOX is a regulator of polyamine metabolism and may be involved in neurodegenerative diseases.	uORF
*OSBP2*	Oxysterol-binding protein 2	2.2	3.3E−8	OSBP2 is involved in lipid transport. It binds to oxysterols and may inhibit their cytotoxicity. It is essential for cell proliferation and survival.	uORF
*GPX3*	Glutathione peroxidase 3	2.4	2.7E−5	GPX3 protects cells and enzymes from oxidative damage.	uORF
*RAP1GAP*	RAP1 GTPase activating protein	4.0	0.0098	RAP1GAP is the most prominent GTPase-activating protein in brain. It is involved in neuronal differentiation.	uORF
*HEY1*	Hes-related family bHLH transcription factor with YRPW motif 1	2.0	0.012	HEY1 is a transcriptional repressor in the Notch signaling pathway which plays an important role in gliogenesis, cardiac morphogenesis and angiogenesis.	uORF
*G0S2*	G0/G1 switch 2	2.6	0.012	G0S2 promotes apoptosis.	uORF
*GCAT*	Glycine C-acetyltransferase	2.0	0.015	GCAT is the second mitochondrial enzyme in a 2-step reaction that converts L-threonine to glycine.	uORF
*MAP1B*	Microtubule-associated protein 1B	2.9	0.020	Dominant germline mutations cause periventricular nodular heterotopia (MIM 618918). MAP1B is involved in microtubule assembly, which is an essential step in neurogenesis.	uORF, long 3′UTR, AS
*SYNM*	Synemin	2.0	0.033	SYNM plays an important cytoskeletal role within the muscle cell cytoskeleton. SYNM is a candidate gene for dilated cardiomyopathy.	uORF, long 3′UTR
*ZNF155*	Zinc finger protein 155	−2.3	6.1E−10	ZNF155 may be involved in transcriptional regulation.	uORF
*OLFM1*	Olfactomedin 1	−2.4	0.012	OLFM1 contributes to the regulation of axonal growth in the embryonic and adult central nervous system.	uORF
*RAVER2*	Ribonucleoprotein, PTB-binding 2	−2.3	0.020	RAVER2 may bind single-stranded nucleic acids.	uORF, long 3′UTR, AS
*NTSR1*	Neurotensin receptor 1	−2.0	0.023	NTSR1 mediates the multiple functions of neurotensin in the brain.	uORF long 3′UTR
*TUBB2A*	Tubulin beta 2A class IIa	−2.9	0.050	TUBB2A is the major constituent of microtubules. Dominant germline mutations cause cortical dysplasia (MIM 615763).	

Abbreviations: *uORF* predicted translated upstream open-reading frame, *UTR* long 3′untranslated region (>1.5 kb), 3′UTR intron, *AS* alternative splicing.

The ranked list enrichment analysis of the DEGs using the GO function (Supplementary Figs 5, 6) revealed enrichments in biological processes involved in the cellular response to toxic substances (adjusted *p*-value calculated using the FGSEA-multilevel method [20], or padj, of 0.025), detoxification (padj = 0.03), the hydrogen peroxide metabolic process (padj = 0.03), and cellular oxidant detoxification (padj = 0.05), suggesting that dysfunctional SMG9 may impair the normal function of the SMG1 complex involved in the cell stress response, and the genotoxic and oxidative stress pathway.

We compared the DEGs between our dataset and a previously published SMG8 and SMG9 deficient patients’ dataset (Alzahrani et al.). There were 20 overlapping DEGs in both datasets: *ZNF155, PRDX6, CDC42EP2, PITHD1, EREG, GABARAPL1, FBXO9, BAG1, IER3, PTGS2, MPZL1, CPEB2, DUSP2, RNF187, KYNU, HECW2, PLSCR1, CNTNAP3, HIF1A*, and *TMEM164* (Supplementary Table 7).

## Discussion

In this study, we report five patients from three unrelated families with a novel homozygous *SMG9* c.551T > C p.(Val184Ala) variant. The ID segregates recessively in the families. The clinical phenotype of all five patients was recognizably similar, and the variant was not present in a homozygous state in the healthy controls of either our own in-house set of Finnish sequencing data or the largest public database of sequence data to date (gnomAD), providing additional support for its role in pathogenicity.

RNA sequencing showed that the *SMG9* c.551T > C variant did not affect the splicing of SMG9 gene products, and the expression levels of SMG9 gene products did not significantly differ between *SMG9* c.551T > C homozygous patients and healthy controls. Allele-specific expression analysis did not provide evidence that the *SMG9* variant affects the PTC-induced NMD mechanism. However, DGE analysis revealed 112 statistically significant DEGs between the cases and controls, with an overall pattern of an increased expression of genes in the patient group. This suggests that normal SMG9 function may have an inhibitory effect on gene expression and the *SMG9* c.551T > C variant causes transcriptional upregulation.

SMG9 is a regulatory cofactor that binds to the SMG1 kinase, which carries out an indispensable phosphorylation step in the NMD pathway [27]. SMG9 is ubiquitously expressed in the brain, heart, eye, and blood [28]. It contains 14 exons and has only one known functional mRNA isoform (NM_019108.4). The *SMG9* gene product, SMG9, contains two functional domains: the N-terminally located intrinsically disordered domain and the C-terminally located nucleotide-trisphosphatase domain [29]. The *SMG9* c.551T > C variant is located in exon 5 of the *SMG9* gene and in the nucleotide-binding G-fold domain of the SMG9 protein, where it faces the active kinase site of SMG1 [30]. As the *SMG9* c.551T > C variant is predicted to be damaging, it possibly reduces the kinase activity of the SMG1.

NMD is a regulatory pathway that functions not only to degrade transcripts containing PTCs, but also to maintain normal transcriptome homeostasis [4, 6]. RNA sequencing results of samples from patients with homozygous *SMG9* c.551T > C and controls showed that 87% (97) of the 112 significantly DEGs were upregulated, suggesting that normal SMG9 function may downregulate transcription of these genes. This is consistent with previous studies that demonstrated a prevalent upregulation of gene expression as a result of SMG9 deficiency [2] and depletion of UPF1, the key protein of NMD, in human embryonic stem cells (hESCs) [31]. In this study, twenty-two of the statistically significant DEGs (*N* = 22/112, 20%) were known NMD substrates upregulated or downregulated in UPF1*-*depleted hESCs (Supplemental Table 6) [31] Our results are consistent with previous studies, which have suggested that constitutional defects in the *SMG8* and *SMG9* genes may cause NMD-related transcriptional dysregulation without affecting PTC-induced NMD [7].

In addition to the NMD pathway, the SMG1 complex plays a role in cell growth, the cell stress response, the genotoxic and oxidative stress pathway, and TNFα-induced apoptosis, which could explain the enrichment of DEGs involved in the cellular response to oxidative damage, toxic substances, detoxification, and apoptosis (Table 2 and Supplementary Fig. 5). Several highly significant DEGs are involved in various pathways, suggesting that multiple processes could play a role in disease pathogenesis.

DGE analysis revealed a number of DEGs which are highly expressed in the brain, heart, and/or eye, which could contribute to disease pathogenesis (Table 2 and Supplementary Table 6). The expression of spermine oxidase (SMOX), a highly inducible enzyme that regulates polyamine metabolism, was significantly upregulated in patients. SMOX-associated dysregulation of polyamine metabolism has been suggested to play a role in neurodegenerative diseases [32, 33], rendering SMOX a biologically interesting candidate in the pathogenesis of these patients’ disease. In addition, elevated SMOX levels and the resultant disturbance of polyamine levels increase the severity of seizures in mice models [33], and overexpression of SMOX is associated both with excitotoxic injury and higher oxidative stress [34]. Other significantly upregulated genes were those that encode: oxysterol-binding protein 2 (OSBP2), which is essential for cell proliferation and survival [35], RAP1 GTPase activating protein (RAP1GAP), which is involved in neuronal differentiation [36], and glutathione peroxidase 3 (GPX3), which protects cells from oxidative damage. Another interesting, upregulated gene was *HEY1*, a transcriptional repressor in the Notch signaling pathway that plays an important role in gliogenesis and cardiac morphogenesis and angiogenesis [37, 38], which could contribute to heart and brain pathogenesis. On the other hand, the expression of zinc finger protein 155 (ZNF155), which may be involved in transcriptional regulation, was significantly downregulated. However, these results should be validated in future studies by confirming the findings using RT-qPCR.

The *SMG9* gene variant c.551T > C is enriched in the Finnish population, and its allele frequency is 34 times higher in Finns (0.0016) than in non-Finnish Europeans (4.6e-05) in the gnomAD. The genetic architecture of the Finnish population is characterized by recent bottlenecks and genetic drift causing enrichment of unique rare variants, some of which are deleterious [23]. This has led to the identification of numerous recessively inherited pathogenic founder variants that are more common in Finns than in any other population, as exemplified in the Finnish Disease Heritage database [22]. It is likely that more novel pathogenic recessive variants will be identified in the Finnish population in future studies.

Recently, seven patients with biallelic loss-of-function variants in *SMG9* were described as having heart and brain malformation syndrome [2, 8–10]. In our cohort of five patients with the same novel homozygous *SMG9* c.551 T > C variant, variable phenotypic penetrance was noted for heart and brain malformations, and consistent findings of ID, pyramidal signs, and dyspraxia were noted in all patients. Several patients also had vertical strabismus, which has been suggested to be part of a broader motor control deficit [39]. All the patients were adults with good general health, providing evidence that the homozygous *SMG9* c.551T > C variant was not significantly associated with reduced life expectancy. The milder phenotype in patients with homozygous damaging *SMG9* c.551T > C missense variant, compared to the phenotypes in the previously reported patients with biallelic loss-of-function variants, suggests that *SMG9* c.551T > C could be a hypomorphic allele resulting in milder developmental outcome.

In conclusion, this study shows that the phenotype of heart and brain malformation syndrome ranges from a characteristic set of heart and brain anomalies to the presentation of ID, pyramidal tract defect, and ocular manifestations extending the knowledge of phenotypic spectrum. RNA sequencing results revealed prevalent upregulation of genes, suggesting that normal SMG9 function is involved in transcriptional downregulation. A series of highly significantly DEGs were identified, including *SMOX, OSBP2, GPX3*, and *ZNF155*; these are candidate genes possibly contributing to the disease pathogenesis. This study and previous studies [1–3, 7] confirm the presence of a novel, emerging clinical group of developmental syndromes caused by pathogenic germline variants in genes encoding components or regulators of NMD machinery. As there are several genes in the NMD pathway that have not yet been associated with human disease, it is possible that novel disease genes in this pathway will be identified in the future.

## Supplementary information

SMG9 supplement

In silico structural modeling of SMG9-Val184Ala

Supplementary Table 6

Supplementary Table 7

## Data Availability

De-identified materials, data sets, and protocols are available upon request. The reported variant was submitted to the LOVD database hosted at Leiden University Medical Center, the Netherlands.
