# Supplementary material for: A novel variant in SMG9 causes intellectual disability, confirming a role for nonsense-mediated decay components in neurocognitive development
Source: Eur J Hum Genet. 2022 Jan 28;30(5):619–27. doi: 10.1038/s41431-022-01046-5 (PMC9090808; doi:10.1038/s41431-022-01046-5)
Supplement: Supplementary file 1 — SMG9 supplement [file 41431_2022_1046_MOESM1_ESM.docx]

**SUPPLEMENTARY MATERIAL**

**A novel variant in *SMG9* causes intellectual disability, confirming a role for nonsense-mediated decay components in neurocognitive development**

Elisa Rahikkala^1,2^, Lea Urpa^3^, Bishwa Ghimire^3^, Hande Topa^4^, Mitja I. Kurki^3,5,6^, Maryna Koskela^4^, Mikko Airavaara^4,7^, Eija Hämäläinen^3^, Katri Pylkäs^8^, Jarmo Körkkö^9^, Helena Savolainen^9^, Anu Suoranta^3^, Aida Bertoli-Avella^10^, Arndt Rolfs^10,11^, Pirkko Mattila^3^, Mark Daly^3,5,6,12,13^, Aarno Palotie^3,5,6,12,13^, Olli Pietiläinen^4,5,14^, Jukka Moilanen^1^, Outi Kuismin^1,3^

Author affiliations

^1^ Department of Clinical Genetics, PEDEGO Research Unit and Medical Research Center Oulu, Oulu University Hospital and University of Oulu, Oulu, Finland

^2^ Institute of Biomedicine, University of Turku, Turku, Finland

^3^ Institute for Molecular Medicine Finland (FIMM), University of Helsinki, Helsinki, Finland

^4^ Neuroscience Center, Helsinki Institute of Life Science, University of Helsinki, Helsinki, Finland

^5^ Psychiatric & Neurodevelopmental Genetics Unit, Massachusetts General Hospital, Boston, MA, USA

^6^ The Stanley Center for Psychiatric Research, The Broad Institute of MIT and Harvard,

Cambridge, MA, USA

^7^Division of Pharmacology and Pharmacotherapy, Faculty of Pharmacy, University of Helsinki

^8^ Cancer and Translational Medicine Research Unit and Biocenter Oulu, University of Oulu, NordLab Oulu, Oulu, Finland

^9^ Northern Ostrobothnia Hospital District, Center for Intellectual Disability Care, Oulu, Finland

^10^ Centogene GmbH, 18055 Rostock, Germany

^11^ Medical Faculty, University of Rostock, Rostock, Germany

^12^ Analytic and Translational Genetics Unit, Department of Medicine, Massachusetts General Hospital, Boston, MA, USA

^13^ Department of Neurology, Massachusetts General Hospital, Boston, MA, USA

^14^ Department of Stem Cell and Regenerative Biology, Harvard University, Cambridge, MA, USA

Table of Contents

[Supplemental clinical data of patients with the homozygous *SMG9* c.551T>C p.(Val184Ala) variant 4](#_Toc89199014)

[**Patient 1** 4](#_Toc89199015)

[**Patient 2** 6](#_Toc89199016)

[**Patient 3** 7](#_Toc89199017)

[**Patient 4** 9](#_Toc89199018)

[**Patient 5** 10](#_Toc89199019)

[Supplementary methods 14](#_Toc89199020)

[**Whole exome sequencing** 14](#_Toc89199021)

[**RNA sequencing** 16](#_Toc89199022)

[Samples 16](#_Toc89199023)

[Supplementary Table 1 16](#_Toc89199024)

[RNA extraction, NGS library preparation and sequencing 16](#_Toc89199025)

[**RNA sequencing data analysis** 17](#_Toc89199026)

[Differential gene expression analysis 17](#_Toc89199027)

[Pathway analysis 18](#_Toc89199028)

[Effects of the SMG9 c.551T>C variant on RNA splicing and gene expression 18](#_Toc89199029)

[**Allele Specific Expression Analysis** 19](#_Toc89199030)

[**In silico assessment of SMG9-Val184Ala** 20](#_Toc89199031)

[**Western blot analysis of SMG9 expression** 20](#_Toc89199032)

[Supplemental Results 22](#_Toc89199033)

[**Prevalence of *SMG9* c.551T>C in ID population versus general population** 22](#_Toc89199034)

[Supplementary Table 2 22](#_Toc89199035)

[**In silico assessment of the SMG9-Val184Ala mutated structure** 23](#_Toc89199036)

[**Western blot** 23](#_Toc89199037)

[**RNA sequencing** 24](#_Toc89199038)

[Supplementary Table 3 24](#_Toc89199039)

[Supplementary Table 4. 25](#_Toc89199040)

[Supplementary Figure 2. 26](#_Toc89199041)

[**Effects of the identified *SMG9* c.551T>C p.(Val184Ala) variant on RNA splicing and gene expression** 27](#_Toc89199042)

[Supplementary Table 5 27](#_Toc89199043)

[**Allele Specific Expression Analysis** 28](#_Toc89199044)

[Supplementary Figure 3. 29](#_Toc89199045)

[Supplementary Figure 4. 31](#_Toc89199046)

[**Differential gene expression analysis** 32](#_Toc89199047)

[**Ranked list enrichment analysis** 32](#_Toc89199048)

[Supplementary Figure 5 33](#_Toc89199049)

[Supplementary Figure 6. 34](#_Toc89199050)

## **Supplemental clinical data of patients with the homozygous *SMG9* c.551T>C p.(Val184Ala) variant**

**Patient 1** (Figure 2, Family 1, II-1) was a 25-year-old man and the first child of healthy non-consanguineous Finnish parents. He was born at gestational week 39, and the delivery was normal. His mother had gestational diabetes; otherwise, the pregnancy was normal. His birth weight (BW) was 3,590 g, birth length (BL) was 51 cm, and occipitofrontal head circumference (OFC) was 35.5 cm. Apgar points at 1/5/10 minutes were 7/9/8.

His motor development was mildly delayed. He learned to walk at the age of 15 months, and at that time, he tended to fall or stumble frequently. His speech development, especially his expressive language, was markedly delayed. He was referred to a speech therapist at the age of 2 years due to lack of speech and started to receive speech therapy. At the age of 2 years and 10 months, he could say only a couple of words, and he used vocalizing, pictures, gestures, and signs to express himself. He had severe oral motor dyspraxia and difficulties producing understandable words. At the age of 4 years, he could express himself by making simple sentences using sign language, but his own speech was limited and unclear. He received intensive speech therapy and gradually started to speak sentences. At the age of 7 years, he received an oral motor exerciser device to treat his motor speech disorder.

At the age of 6 years, occupational therapy was started due to motor clumsiness, concentration problems, and difficulties in eye-hand coordination and visual and spatial perception.

His growth was normal. At the age of 2 years, he had recurrent secretory otitis media infections. He had alternating esotropia, hypertropia in his left eye, and hypotropia in his right eye. Eye patching was used daily, but he failed to develop binocular vision. At the age of 5 years, several strabismus surgeries of his left eye were undertaken, including retropositioning of the inferior oblique muscle, retropositioning and hypoponation of the left medial rectus muscle, and resection and hypoponation of the left lateral rectus muscles. His visual acuity was 1.0/1.0.

A cerebral MRI at the age of 3 years revealed a mild lack of white matter periventricularly in the trigonum area and mild dilatation of the lateral ventricles.

In a psychological assessment at the age of 21 years (WAIS-IV), his cognitive skills corresponded to mild intellectual disability (ID). He had marked word-finding difficulties, and his linguistic processing was slow and arduous. Social skills were his strength. He could read and write.

At the time of this study, he lived independently, and he managed his daily living tasks independently. He was working in supported employment.

His physical health was good, and he was not taking any regular medication. He had mild intention tremor and bradydiadochokinesis. The muscle tone in his lower limbs was increased, and his patella tendon reflexes were symmetrically brisk and his Achilles tendon reflexes were clonic. He did not have strabismus. Otherwise, his neurological examination was normal. He had a high palate. Mild 7° scoliosis was measured by a scoliometer. An echocardiogram of his heart showed normal heart structures.

In previous etiological investigations, Fragile-X repeat expansion was normal. Standard chromosome analysis showed a normal male karyotype.

**Patient 2** (Figure 2, Family 2, II-2) was a 56-year-old man who was the second (2/4) child of healthy, non-consanguineous Finnish parents. His mother’s pregnancy and delivery were normal. His BW was 3,550 g, and BL was 52 cm. His motor development was mildly delayed. He started to sit at the age of 1 year and walk at 1 year and 8 months. After learning to walk, he was still clumsy and often falling down. His language development was markedly delayed. He started to say words at the age of 3 years, and at the age of 5 years, he could say only about 20 words. Due to markedly delayed speech development and unclear speech, he was referred to further investigations and speech therapy at the age of 6 years. In the psychological assessment at the age of 6 years, his cognitive skills corresponded to mild ID. His hearing was normal. Orchidopexy of the right testicle was performed at the age of 6 years, at which age he also had a secretory otitis media infection of his left ear. At the age of 16 years, he suffered from bronchitis, which was treated with oral antibiotics.

He started in normal school at the age of 8 years. He had poor concentration. At the age of 9, he was transferred to special education due to learning problems. His speech was unclear and cluttering.

In an ophthalmological assessment at the age of 8 years, his eyes were normal. His visual acuity was 1.0/1.0. He had no strabismus. At the age of 8 years, he had mild action tremor in his hands, especially when he was performing tasks requiring accuracy. He also had clumsiness in fine motor and coordination skills. He had peripheral ataxia. His hearing was normal. The mobility of his tongue was stiff. Achilles tendon reflexes were symmetrically clonic, and patella reflexes were brisk.

In a psychological assessment at the age of 10 years, his cognitive skills corresponded to borderline moderate/mild ID. The Bender Visual Motor Gestalt test showed distortion and non-appearance of figures. He was social and co-operative.

In the clinical examination of this study, at the age of 56 years, he was interactive and could speak fluently. He had no strabismus, and his eye movements were normal. He had spasticity in his lower limbs, and his Achilles tendon and hamstring muscles were tight. His Achilles tendon reflexes were clonic, and his patella tendon reflexes were very brisk. The Babinski sign was bilaterally negative. He had intention tremor. Diadochokinesis and fine finger movements were slow and clumsy. He had mild difficulties in fine motor skills and co-ordination. He took sidesteps in tandem walking. He had high blood pressure and hypercholesterolemia. He regularly used enalapril and simvastatin medications. His general health was good. He lived independently in a supported living facility where he received a warm meal daily; otherwise, he managed his daily living tasks independently. He was working in supported employment as a caretaker in property maintenance. He could read and write.

In the standard chromosome analysis, benign pericentric inversion was noted; otherwise, he had normal chromosomes. His karyotype was 46,XY,inv(9)(p11q13). At the age of 6 years, his skull x-ray was normal, and an EEG showed spike and slow-wave complexes and once bilateral spike and slow-wave discharge without clinical seizures while sleeping.

**Patient 3** (Figure 2, Family 2, II-3), a 54-year-old man, a brother of Patient 2 and the third child of the family. His mother’s pregnancy and delivery were normal. His BW was 3,450 g. He had a global developmental delay. He started to walk at the age of 15 months and said his first words at the age of 3.5 years. As a child, he was clumsy and often stumbling and falling.

He had had alternating exotropia since birth. At the age of 5 years, strabismus surgeries of both eyes were undertaken, including retropositioning of the lateral rectus muscles. His visual acuity was 1.2/1.2. His hearing was normal. At the age of 4 years, he had bilateral otitis media infections. At the age of 14 years, he suffered from pneumonia that was treated with oral antibiotics.

At the age of 4 years, he received speech therapy due to markedly delayed speech development. He had sound errors in his speech. At the age of 6 years, he could speak short understandable sentences. He started school at the age of 8 years in a special education class. In a psychological assessment at the age of 9 years, his cognitive skills corresponded to moderate ID. His gait was described as ataxic, and in childhood, he already had spasticity in his lower limbs.

In the clinical examination in this study, at the age of 54 years, he was co-operative and could talk fluently, but he appeared to have limited verbal output. He had alternating exotropia in his eyes. He had increased muscle tone in his lower limbs, his patella reflexes were bilaterally brisk, and his Achilles tendon reflexes clonic. The muscle tone and reflexes in his upper limbs were normal. He had intention tremor and balance and coordination problems. He could tandem walk with support. He was not able to walk on his tiptoes or heels. His fine motor skills were also clumsy, and he had bradydiadochokinesis. He had pronounced thoracal kyphosis in his back. He lived in an assisted-living facility, and he was working in sheltered employment. He could read and copy print text from a model.

He had high blood pressure, type II diabetes, and benign prostatic hyperplasia. He regularly used telmisartan, metformin, dutasteride, and tamsulosin medications.

An EEG showed a bitemporal symmetric spike and slow-wave discharges without clinical seizures. Urine amino acid analysis was normal. In the standard chromosome analysis, benign pericentric inversion was noted; otherwise, he had normal chromosomes. His karyotype was 46,XY,inv(9)(p11q13).

**Patient 4** (Figure 2, Family 3, II-1) was a 29-year-old man and the first child of healthy, non-consanguineous Finnish parents. He was born at gestational week 38+6. His BW was 3,270 g, his BL was 50 cm, and his OFC was 35.5 cm. Apgar points at 1/5 minutes were 8/8. Just after birth, he was diagnosed with a congenital heart defect: transposition of the great arteries, ventricular septal defect, atrial septal defect, patent ductus arteriosus, and hypoplastic right atrium. At the age of 1 week, he had arterial switch, atrial septal defect, ventricular septal defect, and patent ductus arteriosus surgery. He developed pulmonary stenosis, which was treated with homograft surgery at the age of 6 months. Homograft replacement surgeries were performed twice.

At the age of 2 years adenoidectomy and tympanostomy tube placement were performed due to recurrent secretory otitis media infections. His hearing was normal. He had strabismus in his right eye. His growth was normal.

Muscular hypotonia was noted in infancy, and physiotherapy started at the age of 10 months. He learned to walk at the age of 1 year and 6 months. He had mild balance problems and clumsiness. His language development was markedly delayed. He said his first words at the age of 2.5 years. Speech therapy was started at the age of 3 years. He had facial hypotonia, oral motor dyspraxia, and unclear speech. He used gestures to facilitate communication. He had dyspraxia and intention tremor in his hands, and occupational therapy was started at the age of 4 years.

In a developmental assessment at the age of 16 years (WISC-III), his cognitive skills corresponded to moderate ID. He could read and write. He had tremor that impaired performing tasks that required fine motor skills (e.g., tying shoelaces or fastening buttons).

In the clinical examination in this study, at the age of 26 years, he was social and interactive. He had a hoarse voice. He had exotropia and hypertropia in his right eye. His palate was high. He had an intention tremor and bradydiadochokinesis. He had mild spasticity in his legs and tight Achilles tendons. He had symmetrically clonic Achilles tendon reflexes and very brisk patella and brachioradialis tendon reflexes. The Babinski signs were negative. He had planovalgus in his feet and a sandal gap in his left foot. He had brachycephaly.

Cerebral MRI scans at the ages of 3 and 6 years were normal. Skull x-ray at the age of 7 years showed brachycephalic skull. At the age of 4 years, his bone age was normal. Standard chromosome analysis and Fragile-X repeat expansion were normal.

**Patient 5** (Figure 2, Family 3, II-2) was a 26-year-old man, the brother of Patient 4, and the second (2/3) child of healthy, non-consanguineous Finnish parents. He was born at gestational week 38+6, and the delivery was normal. His BW was 2,535 g, his BL was 46 cm, and his OFC was 32 cm. Apgar points at 1/5/10 minutes were 8/9/9. He had constipation and poor weight gain in infancy. He had short stature.

He had recurrent secretory otitis media infections, which were treated with antibiotics, adenoidectomy, and repeated tympanostomy tube insertions. A tonsillectomy was performed at the age of 5 years due to hypertrophic palatine tonsils and snoring. His hearing was normal.

At the age of 1 year, he was referred to the pediatric neurology department due to muscular hypotonia. He had a global developmental delay. He learned to walk at the age of 1 year and 7 months. As a child, he was clumsy, had poor balance and coordination, and was often falling down. He was also described as ataxic as a child.

His speech development was markedly delayed. At the age of 3 years and 4 months, he could say only a couple of words, but he could understand simple speech. Speech therapy was started. At the age of 5 years, he was communicating using signs and speech, which was still limited, unclear, and dysphasic. He received intensive speech therapy until the age of 10 years, and he gradually learned to speak intelligible sentences.

Occupational therapy was started at the age of 5 years to improve his gross and fine motor skills, bilateral hand use, spatial perception, and visuomotor and social skills. He also had concentration problems, dyspraxia, and eye-hand coordination problems.

In a developmental assessment at the age of 16 years (WISC-III), his cognitive skills corresponded to moderate ID. He could read and write simple text.

In an ophthalmological assessment, he had alternating exotropia in both eyes and hypertropia in his right eye when he was looking into the distance. At the age of 15 years, strabismus surgery of his right eye was undertaken, including retropositioning of the superior rectus muscle. After the operation, he had exophoria in his right eye.

In the clinical examination in this study, at the age of 26 years, he was interactive, positive, and spoke in sentences. He had mild hypotonia in his face, an inability to pronounce the alveolar trill, and mild word finding problems. He had exotropia and hypertropia in his right eye. His palate was narrow and high, and his upper dental arch was cramped. He had mild intention tremor and bradydiadochokinesis. The muscle tone in his legs was mildly increased. He had symmetrically clonic Achilles tendon reflexes and very brisk patella and brachioradialis tendon reflexes. The Babinski signs were negative. He had bilateral mild planovalgus. He had pronounced lumbar lordosis. He had a sacral dimple. His visual acuity was 1.0/1.6. He had a prominent forehead, depressed and wide nasal bridge, broad nasal tip, low insertion of columella, high and narrow palate, and brachydactyly.

A cerebral MRI scan at the age of 3 years was normal. An EEG at the age of 5 years showed slow background activity and, during sleep, a couple of spike and slow-wave complexes without clinical seizures. At the age of 4 years, his bone age corresponded to that of a 5-year-old boy. He had mild clinodactyly and hypoplasia of the middle phalanx of his left fifth finger. Standard chromosome analysis and Fragile-X repeat expansion were normal.

In the developmental assessment at the age of 16 years (WISC-III) his cognitive skills corresponded to moderate ID. He could read and write. He had tremor impairing tasks requiring fine motor skills e.g. tying his shoelaces or buttoning the buttons.

In the clinical examination at the age of 26 years he was social and interactive. He had a hoarse voice. He had exotropia and hypertropia in his right eye. His palate was high. He had intention tremor and bradydiadochokinesis. He had mild spasticity in his legs and tight Achilles tendons. He had symmetrically clonic Achilles tendon reflexes and very brisk patella and brachioradialis tendon reflexes. Babinski signs were negative. He had planovalgus in his feet and a sandal gap in his left foot. He had brachycephaly.

A cerebral MRI at the age of 3 and 6 years was normal. Skull x-ray at the age of 7 years showed brachycephalic skull. At the age of 4 years his bone age was normal. Standard chromosome analysis and Fragile-X repeat expansion was normal. Further, methylation studies for Angelman syndrome test showed the presence of both maternal and paternal chromosomes in the 15q11-q13 area. Array-CHG was normal.

**Supplementary methods**

### **Whole exome sequencing**

Whole exome sequencing (WES) of DNA samples from Patients 1-3 and Control 3, data analysis, and annotation were performed as previously described at the Broad Institute, USA (1,2). Briefly, the exome was captured with the Nextera Rapid Capture Exome Kit (Illumina, San Diego, CA, USA) and the exomes were sequenced using an Illumina HiSeq2500. Two paired-end 100 bp reads were used for WES to provide mean sequence coverage of about 69×, with approximately 85% of the target bases having at least 20× coverage. The sequence reads were mapped to the reference sequence to produce a file in binary alignment map (BAM) format, sorted by coordinate. Variant calling was done by running the HaplotypeCaller in GVCF mode on each sample’s BAM files to create genomic variant call formats (gVCFs). Variants were annotated using VEP v.85 (3) and the LOFTEE VEP plugin (4) to filter out likely false-positive protein truncating variants (PTV). We filtered the variants to high confidence PTV and damaging missense variants (CADD score > 20) and with allele frequency less than 1.0% or absent in the Genome Aggregation Database (gnomAD) v2 and with no reported homozygous carriers in either (gnomAD) (4) or our in-house database of Finnish population control exomes (5). VEP annotation and all other exome sequencing analysis was performed with Hail (Hail Team. https://github.com/hail-is/hail/). Following filtering of the WES data, three patients from two families were identified with a homozygous *SMG9* c.551T>C p.(Val184Ala) (GenBank: NM_019108.4) variant.

The WES of DNA samples from Patients 4 and 5 and Control 2 was performed as described previously (6). In short, the Nextera Rapid Capture Exome Kit (Illumina, San Diego, CA, USA) or the SureSelect Human All Exon kit (Agilent, Santa Clara, CA, USA) were used for enrichment, and a HiSeq4000 (Illumina) instrument for the actual sequencing with the average coverage targeted to 100x. Variants calling, annotation, and prioritization were based on a set of publicly available and in-house tools. WES was performed using DNA samples from the index, parents, and affected sibling. After variant annotations, filtering and prioritization were performed with an in-house developed tool. Trained scientists and human geneticists evaluated the clinical and genetic data. Relevant variants were considered based on compatibility with the suspected phenotype and disease mechanism. All provided clinical data, family history, and available test results were considered. The clinical information was ‘translated’ into human phenotype ontology (HPO) terms, registered in the database, and applied for analysis during variant filtration. For the selected variants, the mode of inheritance of the gene (OMIM®) and all relevant variant information were considered: zygosity, type of variant, and frequency in public databases (gnomAD (4), ExAc (7),) and disease-centered databases (HGMD (8), CentoMD® (9)). Variant nomenclature followed standard recommendations (10). Selected candidate variants were classified according to published ACMG guidelines as pathogenic (P), likely pathogenic (LP), and variant of unknown significance (VUS) (11). Likely benign and benign variants were excluded from reporting. Following filtering of WES data a homozygous *SMG9* c.551T>C p.(Val184Ala) (GenBank: NM_019108.4) variant was identified in both patients. Their parents were found to be heterozygous carriers of this variant.

Confirmation and segregation of the *SMG9* c.551T>C p.(Val184Ala) variant were studied by PCR, followed by conventional Sanger sequencing. Primer sequences and PCR conditions are available upon request.

### **RNA sequencing**

#### Samples

RNA sequencing was performed on RNA samples from all five patients homozygous for the *SMG9* c.551T>C p.(Val184Ala) variant and five age- and sex-matched healthy control individuals. Detailed information about the cases and controls and their *SMG9* c.551T>C genotypes are shown in Supplementary Table 1.

Supplementary Table 1 showing information about the cases and controls. All the cases and controls were males. The average age of patients was 38 years (range 25-56) and the average age of controls was 39.4 years (range 19-58).

| **Patient/control** | **Information** | **Genotype** |
| --- | --- | --- |
|  |  | g.19:43747479A>G (*SMG9* c.551T>C) |
| Patient_1 | Patient 1 | G/G homozygote |
| Patient_2 | Patient 2, brother of Patient 3 | G/G homozygote |
| Patient_3 | Patient 3, brother of Patient 2 | G/G homozygote |
| Patient_4 | Patient 4, brother of Patient 5 | G/G homozygote |
| Patient_5 | Patient 5, brother of Patient 4 | G/G homozygote |
| Control_1 | non-related healthy male control | NA, genotyping not performed |
| Control_2 | healthy control, father of Patients 4 and 5 | A/G heterozygote |
| Control_3 | healthy control, father of Patient 1 | A/G heterozygote |
| Control_4 | non-related healthy male control | A/A wild type |
| Control_5 | healthy male control, maternal cousin of Patients 2 and 3 | A/A wild type |

#### RNA extraction, NGS library preparation and sequencing

Standard methods were used to extract total RNA from the peripheral blood samples of the five probands and five age- and sex-matched healthy control individuals. The quality and quantity of the extracted total RNA were analyzed with a 2100 Bioanalyzer using an RNA 6000 Nano Kit (Agilent, Santa Clara, CA, USA) and a Qubit RNA BR Kit (Thermo Fisher Scientific, Waltham, MA, USA). A Qubit DNA BR kit (Thermo Fisher Scientific, Waltham, MA, USA) was used to measure genomic DNA contamination.

Dual-indexed RNA libraries were prepared from 800 ng of total RNA using the TruSeq Stranded Total RNA library kit (Illumina, San Diego, CA, USA) according to reference guide version 1000000040499v00 Oct 2017. Ribo-Zero Gold (Illumina, San Diego, CA, USA) was used for the depletion of cytoplasmic and mitochondrial rRNA. Sequencing was performed using a NovaSeq 6000 System (Illumina, San Diego, CA, USA). Read length for the paired-end run was 2x101 bp, and the minimum target read was a depth of > 70 M paired-end reads for each library. An in-house pipeline was used for the primary quality evaluation of the RNA sequencing data (12).

### **RNA sequencing data analysis**

#### Differential gene expression analysis

Differential gene expression analysis was performed using edgeR (version 3.28.1) (13) and R [https://www.R-project.org] (version 3.6.0). Raw gene count data were filtered using edgeR’s default parameters and TMM (trimmed mean of M values) normalized. EdgeR’s exact test was used to detect differentially expressed genes (DEGs) in two groups. DEGs were further filtered based on false discovery rate adjusted p-value (FDR padj < 0.05) and log fold-change (logFC) (-0.25 ≥ *logfc* ≥ 0.25) cutoffs.

Differential transcript analysis was performed using Ballgown (14) (version 2.18.0) from transcripts reconstructed using Stringtie (version 1.3.2) (15). Low variance transcripts were filtered, and transcripts were tested for differential expression in two groups using linear models. Differentially expressed transcripts were further filtered based on the q-value cutoff (*qvalue* < 0.05).

The likelihood of differentially expressed genes being more upregulated or downregulated, given the assumption of equal likelihood of being up- or down-regulated, was calculated with a two-tailed binomial test.

All 13,822 protein coding genes from the DEG analysis were used to perform a ranked list enrichment analysis. Neither FDR padj nor fold-change cut-off was used to filter DEGs. This way, even genes with a very small individual effect could be used in identifying overrepresented pathways.

#### Pathway analysis

Molecular Signatures Database (MsigDB) version 7.2 (16), gene sets were used in the analysis. MsigDB has a collection of gene sets and pathways from many popular databases. However, only Gene Ontology (GO), Kyoto Encyclopedia of Genes and Genomes (KEGG), and Reactome pathway database were used in the analysis (17–19).

Genes were pre-ranked based on -log10 (p-value)*sign(logFC). Ranked genes were tested for overrepresented pathways using FGSEA (version 1.16.0) and R (version 4.0.0) using the enrichment score statistic (20). The FGSEA-multilevel method was used to calculate p-values.

#### Effects of the SMG9 c.551T>C variant on RNA splicing and gene expression

The institute for molecular medicine Finland (FIMM) rnaseq2 pipeline was used to process and align the reads, perform the quality check of the data, and calculate the read counts as well as the reads per kilobase per million mapped reads (RPKM) and counts per million (CPM) values. A detailed description of the pipeline can be found in Kumar et. al. (12).

###

### **Allele Specific Expression Analysis**

To determine genes and transcripts likely targeted by the nonsense-mediated decay mechanism, we filtered the exome sequencing data of Patients 1-4 and Controls 2-3 to variants with VEP predicted stop_gain effect on a high-confidence transcript (either designated as canonical, with APPRIS (21) designation P1, P2, or P3, or with transcript support level designation 1). Genotypes were filtered to high confidence genotype calls, with sequencing depth greater than 8 and genotype quality greater than 15, and to heterozygous genotype calls. Sex chromosomes were excluded from analysis.

Based on the findings of Lindeboom et al 2019 (22), we further filtered the high-confidence stop gain (i.e. nonsense, premature truncating codon) variants to those that did not occur in either 1) the last exon, 2) the last 50bp of the second to last exon, 3) the first 150bp of the coding sequence, or 4) in an exon of length greater than 407 nucleotides. Information on presence in last exon or the last 50bp of the penultimate exon (rules 1 and 2) were obtained from the LOFTEE plugin info, information on variant position in the coding sequence was obtained by VEP annotation, and length of exon was obtained via Ensembl (v.85) (23) gene sets.

Allele-specific expression analysis was performed using ASEReadCounter from Genome Analysis Toolkit (version 4.2.0.0-0) on SNPs using default parameters (24). Variants were filtered to those with minimum total read count of at least 5, and proportion of reference reads (reference reads divided by total reads) was calculated for each variant. The proportion of reference reads on average over all individuals was compared between protein truncating variants predicted to be targeted by the NMD mechanism, protein truncating variants predicted to escape NMD, and other variants. To avoid overcounting, as each variant on a transcript is likely to have allele-specific expression if another does, we randomly chose one variant per gene, per individual and tested for a difference between the mean proportion of reference reads of NMD targeted variants and other variants. We repeated this random sampling of one variant per gene 1000 times. Mean proportion was compared between groups with unpaired two-sample Wilcoxon test.

### **In silico assessment of SMG9-Val184Ala**

The crystal structure of SMG9 was obtained from a recently published article that reported the cryo-EM structure of an SMG1-SMG8-SMG9 complex (<http://dx.doi.org/10.2210/pdb6syt/pdb>) (25). SWISS-MODEL was used to predict the structure of the SMG9-Val184Ala mutant (26). The predicted SMG9-Val184Ala mutated structure was superimposed on the wild type SMG9 structure (Figure 3B, Supplemental video).

### **Western blot analysis of SMG9 expression**

Samples of peripheral blood mononuclear cell (PBMC) or human embryonic kidney 293 cells (CRL-1573, ATCC, USA) were resuspended in a lysis buffer (20 mM HEPES, pH 7.4, 100 mM NaCl, 0.5% NP-40, 1.5 mM MgCl_2_, 10% glycerol, 0.5mM DTT) with the addition of protease and phosphatase inhibitors (Roche, Switzerland), incubated on ice for 20 min and centrifuged at 12000g for 20 min at 4ºC. The protein concentration in the supernatants was determined by *DC*™ Protein Assay (Bio RAD, CA, USA), according to the manufacturer’s protocol. The samples containing approximately 30 µg of protein were subjected to 4-12% gradient NuPAGE Bis-Tris gel electrophoresis (ThermoFisher Scientific, USA), according to the manufacturer protocol. Then proteins were transferred to the PVDF membrane using the Novex system (ThermoFisher Scientific, USA). After transfer, the membrane was incubated in blocking buffer (5% w/v nonfat dry milk, PBS, 0.5% Tween-20) for 1 hour to block unspecific binding. The membrane was incubated at 4ºC overnight in the presence of rabbit anti-C19orf61 antibodies (1:1000, PA5-68007, Invitrogen, ThermoFisher Scientific, USA) that recognize SMG9 protein. After washing, the membrane was incubated with horseradish peroxidase-conjugated secondary anti-rabbit (1:3000, NA9340, GE Healthcare, USA) for 1 hour at room temperature. After protein bands detection, the membrane was washed in PBS-T buffer (PBS, 0.5% Tween-20) and re-probed with mouse anti-GAPDH (1:5000, MAB374, Millipore, USA). After washing, the membrane was incubated with horseradish peroxidase-conjugated secondary goat anti-mouse antibodies (1:3000, Dako, Denmark) for 1 hour. The protein bands were detected using a chemiluminescent substrate (Pierce, ThermoFisher, MA, USA).

## **Supplemental Results**

### **Prevalence of *SMG9* c.551T>C in ID population versus general population**

The frequency of the *SMG9* c.551T>C allele in 966 individuals of the Northern Finnish Intellectual Disability Cohort was compared to the general Finnish population, both our own Finnish population controls (the FINRISK and H2000 cohorts) and publicly available population controls (the gnomAD). Statistics calculated with MedCalc (https://www.medcalc.org/calc/odds_ratio.php).

Supplementary Table 2**.** Comparison of allele prevalence in Finnish ID patient versus Finnish population controls. AC = allele count, AN = allele number, AF = allele frequency.

| Control group | ID patient AC | ID patient AN | ID patient AF | Population control AC | Population control AN | Population control AF | OR | 95% CI | P value |
| --- | --- | --- | --- | --- | --- | --- | --- | --- | --- |
| Finnish population controls | 15 | 1932 | 0.007764 | 12 | 12490 | 0.000961 | 8.08 | 3.78 – 17.29 | p<0.001 |
| Gnomad v2 | 15 | 1932 | 0.007764 | 40 | 25038 | 0.001598 | 4.86 | 2.68 – 8,81 | p<0.001 |
| Gnomad v3 | 15 | 1932 | 0.007764 | 9 | 10622 | 0.000847 | 9.16 | 4.00 – 20.97 | p<0.001 |

### **In silico assessment of the SMG9-Val184Ala mutated structure**

Supplementary Figure 1 showing A. Wild type SMG9 binding site B. Mutated SMG9 p.184Ala binding site modelled using SWISS-MODEL. In the predicted model of mutated SMG9 p.184Ala structure, the magnesium ion is not conserved and there is a structural defect in the G domain, which is the ATP binding site.

**
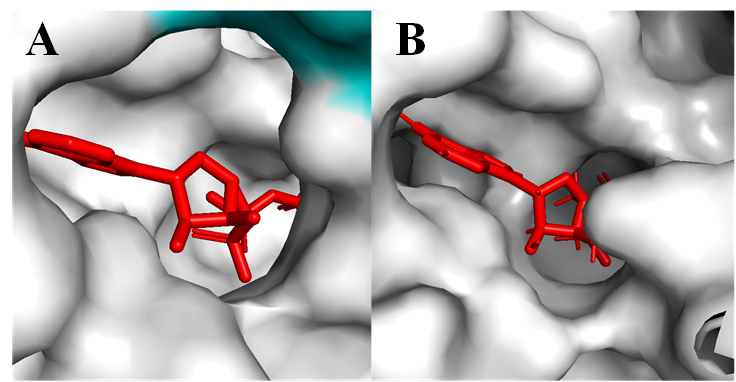
**

### **Western blot**

To test whether the SMG9 protein stability is affected, we performed a western blot analysis. We did not observe the SMG9 expression in control and *SMG9* c.551T>C homozygous patient samples. However, we detected SMG9 expression in HEK cells. The results suggest that either SMG9 protein is not expressed in PBMC or the expression is beyond the detection limit.

### **RNA sequencing**

The technical quality of the RNA sequencing data was good (Supplementary Table 3).

Supplementary Table 3**.** In the sequencing run, at least 70 million PE reads were produced for each sample

| Project | Sample | Reads | Yield (Mb) | Yield (Gb) | Planned Yield (Gb) |
| --- | --- | --- | --- | --- | --- |
| SMG9 | Patient_1 | 192167522 | 38818 | 38,818 | 30 |
| SMG9 | Patient_2 | 152196258 | 30744 | 30,744 | 30 |
| SMG9 | Patient_3 | 138489234 | 27975 | 27,975 | 30 |
| SMG9 | Patient_4 | 160020365 | 32324 | 32,324 | 30 |
| SMG9 | Patient_5 | 166228214 | 33579 | 33,579 | 30 |
| SMG9 | Control_1 | 186344107 | 37642 | 37,642 | 30 |
| SMG9 | Control_2 | 149525611 | 30204 | 30,204 | 30 |
| SMG9 | Control_3 | 163041377 | 32934 | 32,934 | 30 |
| SMG9 | Control_4 | 159828031 | 32285 | 32,285 | 30 |
| SMG9 | Control_5 | 169432535 | 34226 | 34,226 | 30 |

Mb = megabases, Gb = gigabases

The main quality metrics of the RNA sequencing data have been collated into Supplementary Table 4. Target mapping statistics were met for the analysis, and rRNA and intergenic rates were low.

Supplementary Table 4. Mapped reads

| Sample | Mapped | Mapping Rate | Mapped Unique | Mapped Unique | Unique Rate of Mapped | rRNA rate |
| --- | --- | --- | --- | --- | --- | --- |
|  |  |  |  | Rate of Total |  |  |
| Patient_1 | 358,867,285 | 0.979 | 117,146,616 | 0.32 | 0.326 | 0.023 |
| Patient_2 | 268,325,849 | 0.953 | 114,859,942 | 0.408 | 0.428 | 0.013 |
| Patient_3 | 245,122,246 | 0.956 | 122,905,559 | 0.479 | 0.501 | 0.018 |
| Patient_4 | 278,441,167 | 0.956 | 110,091,460 | 0.378 | 0.395 | 0.011 |
| Patient_5 | 297,558,708 | 0.963 | 126,047,870 | 0.408 | 0.424 | 0.012 |
| Control_1 | 350,982,611 | 0.978 | 188,824,746 | 0.526 | 0.538 | 0.017 |
| Control_2 | 263,800,747 | 0.951 | 137,632,014 | 0.496 | 0.522 | 0.016 |
| Control_3 | 292,092,279 | 0.96 | 154,154,594 | 0.507 | 0.528 | 0.02 |
| Control_4 | 287,063,478 | 0.959 | 168,601,142 | 0.563 | 0.587 | 0.015 |
| Control_5 | 303,440,291 | 0.959 | 152,158,793 | 0.481 | 0.501 | 0.013 |

Supplementary Figure 2. Visualization of the alignment RNA data the *SMG9* c.551T>C p.(Val184Ala) variant in exon 5. The visualization was generated using Integrative Genomic viewer (IGV). DNA sample was not available from control 1 individual, but RNA sequencing data demonstrates that this individual is g.19:43747479 A/A.


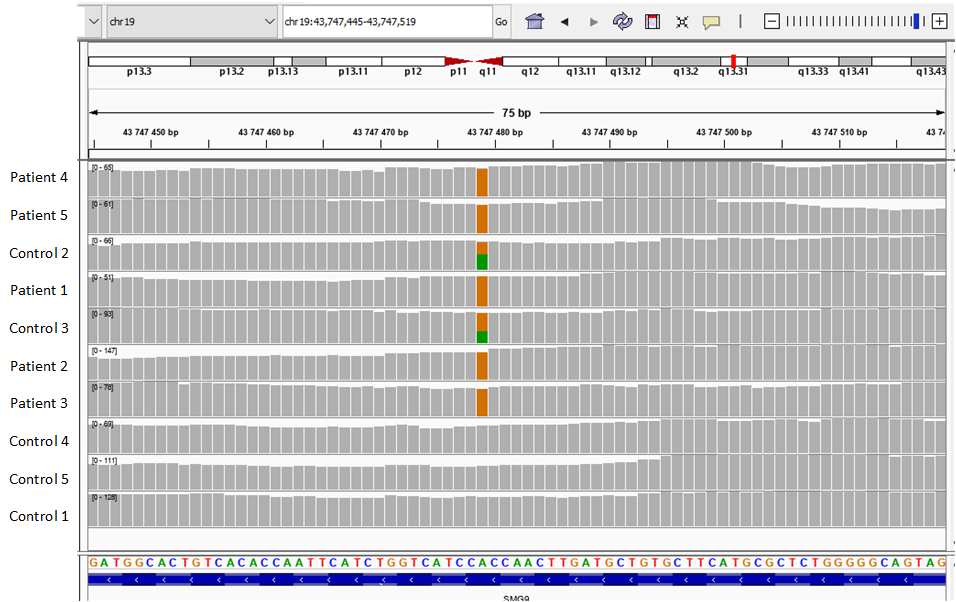


### **Effects of the identified *SMG9* c.551T>C p.(Val184Ala) variant on RNA splicing and gene expression**

Supplementary Table 5. The mean fragments per kilobase million (FPKM) mapped read values of the *SMG9* transcripts in patients and controls. FPKM of the main *SMG9* transcript NM_019108.4, corresponding to Ensembl transcript ENST00000270066, was 0.98 in patients and 1.29 in controls. The differences were not statistically significant (p value > 0.05).

| SMG9 transcript | mean FPKM in patients | mean FPKM in controls |
| --- | --- | --- |
| ENST00000270066 | 0.9833278 | 1.2947318 |
| ENST00000594081 | 0.4513282 | 0.6411446 |
| ENST00000595700 | 0.7205914 | 1.0103558 |
| ENST00000596714 | 0.0093578 | 0.011087 |
| ENST00000597586 | 0.0736786 | 0.1114822 |
| ENST00000597598 | 0.197932 | 0.311884 |
| ENST00000598860 | 0.6872698 | 1.0310136 |
| ENST00000598886 | 0.219617 | 0.3427038 |
| ENST00000599804 | 0 | 0 |
| ENST00000600097 | 0.2021326 | 0.2902288 |
| ENST00000601170 | 0.3604846 | 0.4924436 |
| ENST00000601925 | 0 | 0 |
| ENST00000602222 | 0.00014 | 0.0006266 |

### **Allele Specific Expression Analysis**

Allele-specific expression analysis was performed by randomly choosing one variant per gene from the output of ASEReadCounter, permuting the random choice 1000 times. The results in Figure 3C represent one example of the tests, but all 1000 permutations gave a significantly higher proportion of reference reads in predicted NMD-targeted variants compared to other variants. The statistics of the p values for the 1000 tests were as follows: min 0.002860, mean 0.003615, max 0.004493.

Allele-specific expression analysis was also run on the two control individuals, where we found that there was not a significant difference in proportion of reference reads between likely NMD-targeted variants and other, non-protein-truncating variants. However, previous work has shown that likely NMD-targeted variants determined by the same rules that we applied have a higher proportion of reference reads in a large cohort of unaffected individuals in multiple tissues (27). As in the previous analysis, the test for controls was run by randomly selecting one variant per gene per sample, and repeated 1000 times. The statistics for the p values of 1000 tests for the control were as follows: min 0.2440, mean 0.2873, max 0.3345.

Supplementary Figure 3. Plot comparing the proportion of reference reads from likely NMD-targeted variants compared to non-protein-truncating variants, in Controls 2-3 (left) and Patients 1-4 (right, as seen in Figure 3C). For the Controls 2-3, NMD-targeted variants n=21, other variants n=15585. For Patients 1-4, NMD-targeted variants n=28, other variants n=33913. There was no significant difference between patients and the controls in the proportion of reference reads for NMD-targeted variants (p=0.2103).


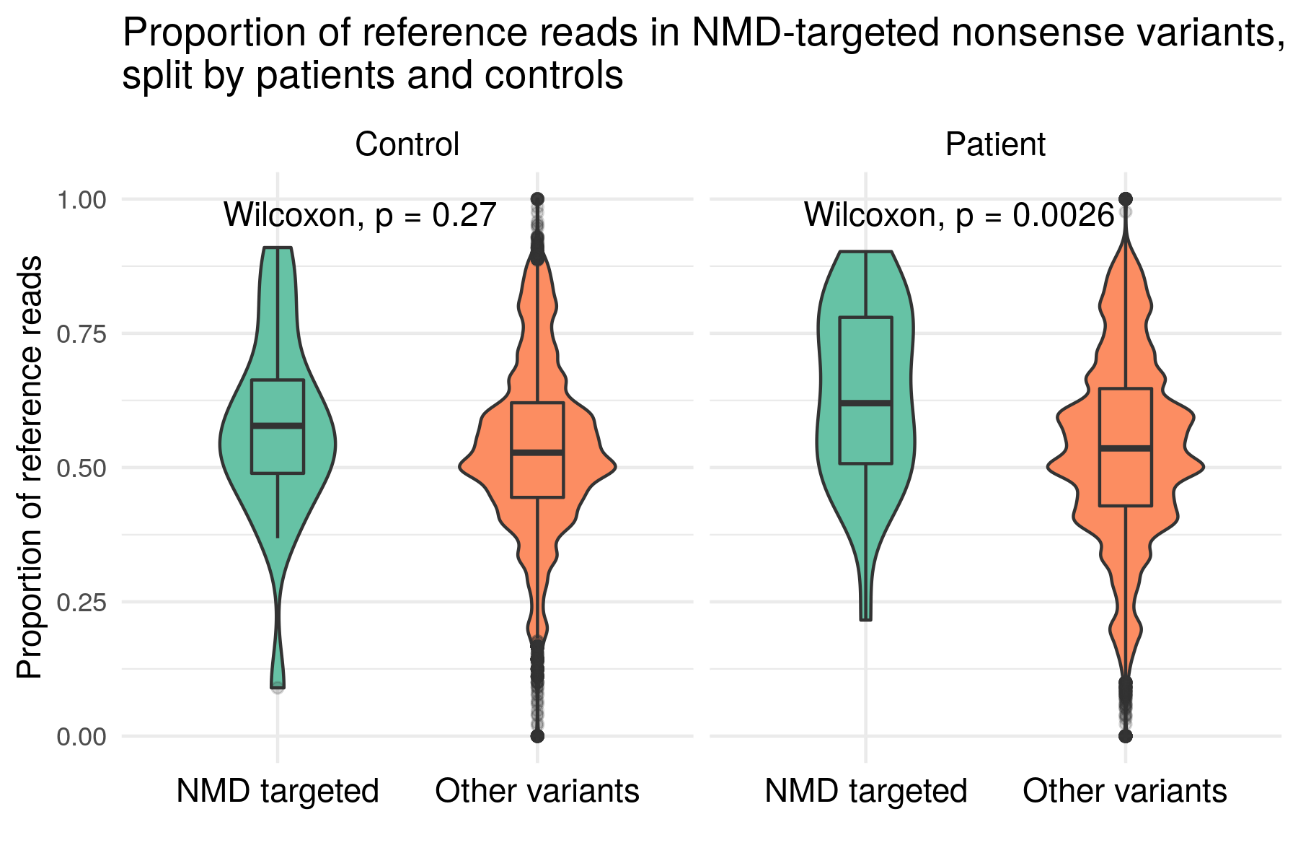


We also compared the proportion of reference reads of predicted NMD-targeted variants to those variants that were predicted NMD-escaping, that is, variants that were VEP annotated as stop_gain but were located either 1) in the last exon of a transcript, 2) in the last 50 base pairs of the second to last exon, 3) in the first 150 base pairs of the coding sequence of the transcript, or 4) in an exon longer than 407 nucleotides. As was found in a recent study (27), the predicted NMD-escaping variants had a lower proportion of reference reads than predicted NMD-targeted variants. Similar to the previous analyses, these tests were run by randomly selecting one variant per gene per sample, and repeated 1000 times. The statistics for the p values in all permutations were as follows. For NMD-escaping variants vs other variants: min 0.1870, mean 0.2047, max 0.2249. For NMD- targeted variants vs other variants: min 0.002782, mean 0.003615, max 0.004648. For NMD-targeted vs NMD escaping variants: min 0.01067, mean 0.01067, max 0.01067.

Supplementary Figure 4. Plot comparing the proportion of reference reads from likely NMD-targeted variants, predicted NMD-escaping variants, and other, non-protein-truncating variants. The proportion of reference reads in likely NMD-targeted variants (n=49) was significantly higher than both predicted NMD-escaping variants (n=18) and other variants (n=49498), and there was no statistically significant difference between predicted NMD-escaping variants and other variants. Groups were compared with Wilcoxon test.


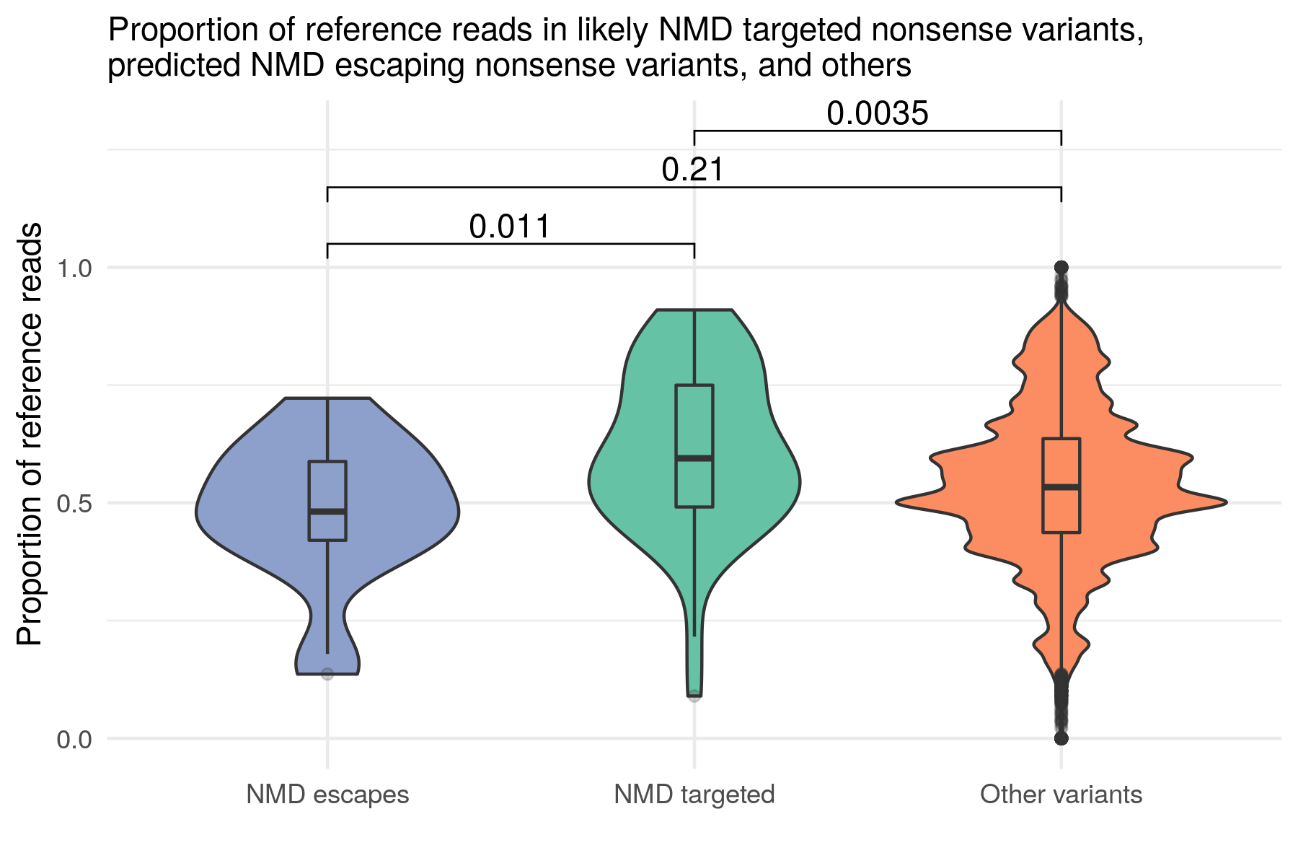


###

### **Differential gene expression analysis**

Among the significantly downregulated DEGs, there were two known disease genes associated with global developmental delay: *TRIM71* (MIM 618570) (logFC = -1.7, FDR padj = 0.02), and *TUBB2A* (MIM 615101) (logFC = -2.9, FDR padj = 0.05). Among the significantly upregulated DEGs, there were four known disease genes associated with global developmental delay: *MAP1B* (MIM 157129) (logFC = 2.9, FDR padj = 0.02)*, HECW2* (MIM 617245) (logFC = 0.9, FDR padj = 0.03)*, PDE4D* (MIM 600129) (logFC = 0.6, FDR padj = 0.04), and *ABHD5* (MIM 604780) (logFC = 0.9, FDR padj = 0.01). The expression of DTNA (MIM 601239), which is associated with congenital heart defects, was decreased in the patients compared with the controls (logFC = -1.3, FDR padj = 0.03).

### **Ranked list enrichment analysis**

Supplementary Figures 5-6 demonstrate the ranked list enrichment analysis of DEGs using Gene Ontology (GO) function. KEGG and Reactome pathway analysis of DEGs did not show statistically significant enrichment of any pathway.

Supplementary Figure 5**.** Demonstrating Gene Ontology (GO) top pathways enrichments in biological processes involved in cellular response to toxic substance (adjusted p-value (padj) 0.025), detoxification (padj 0.03), hydrogen peroxide metabolic process (padj 0.03), cellular oxidant detoxification (padj 0.05) and response to toxic substance (padj 0.05).


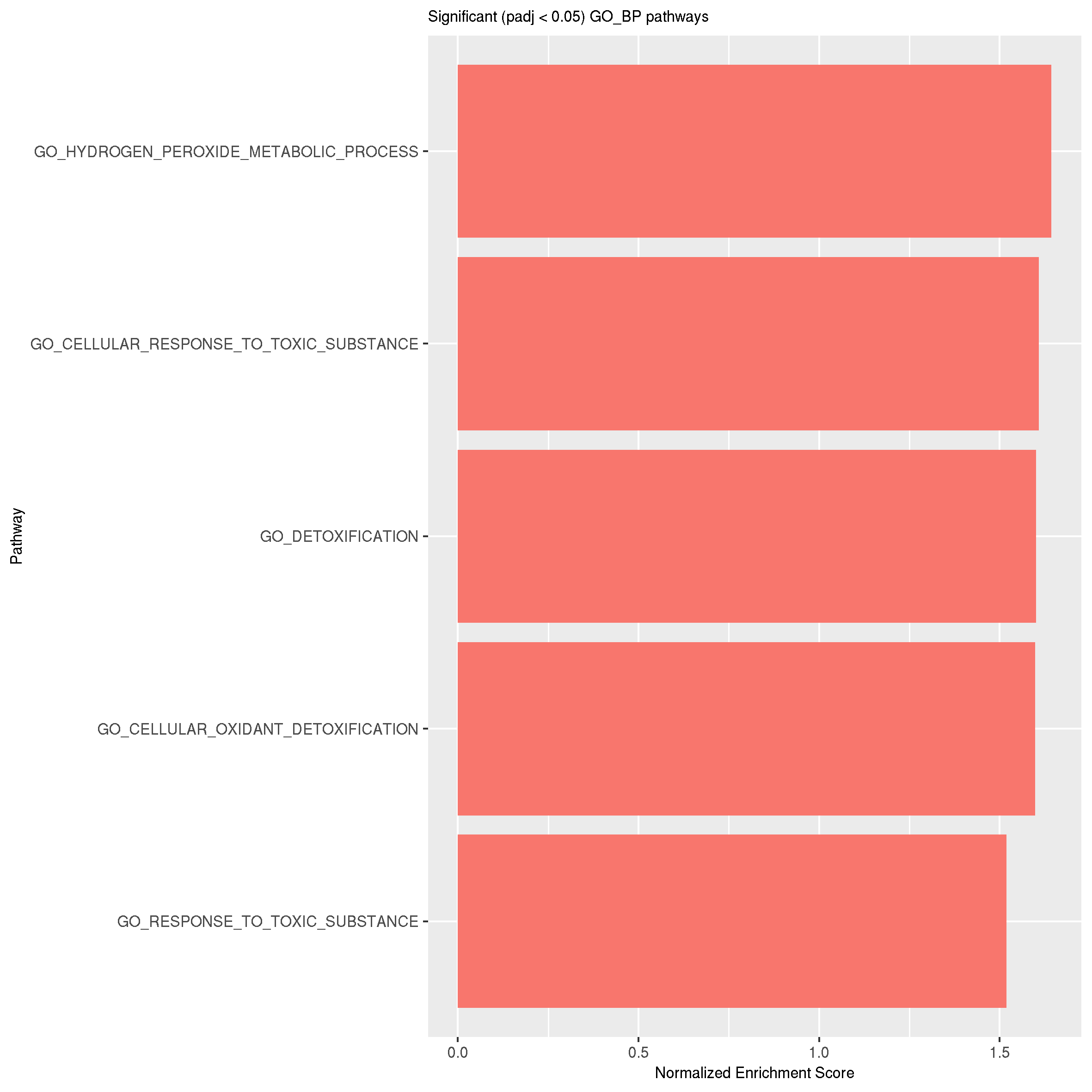


Supplementary Figure 6. Demonstrating Gene Ontology (GO) top pathways of enrichments in GO molecular functions involving antioxidant activity (padj 0.01) and oxidoreductase activity acting on peroxide as acceptor (padj 0.02).


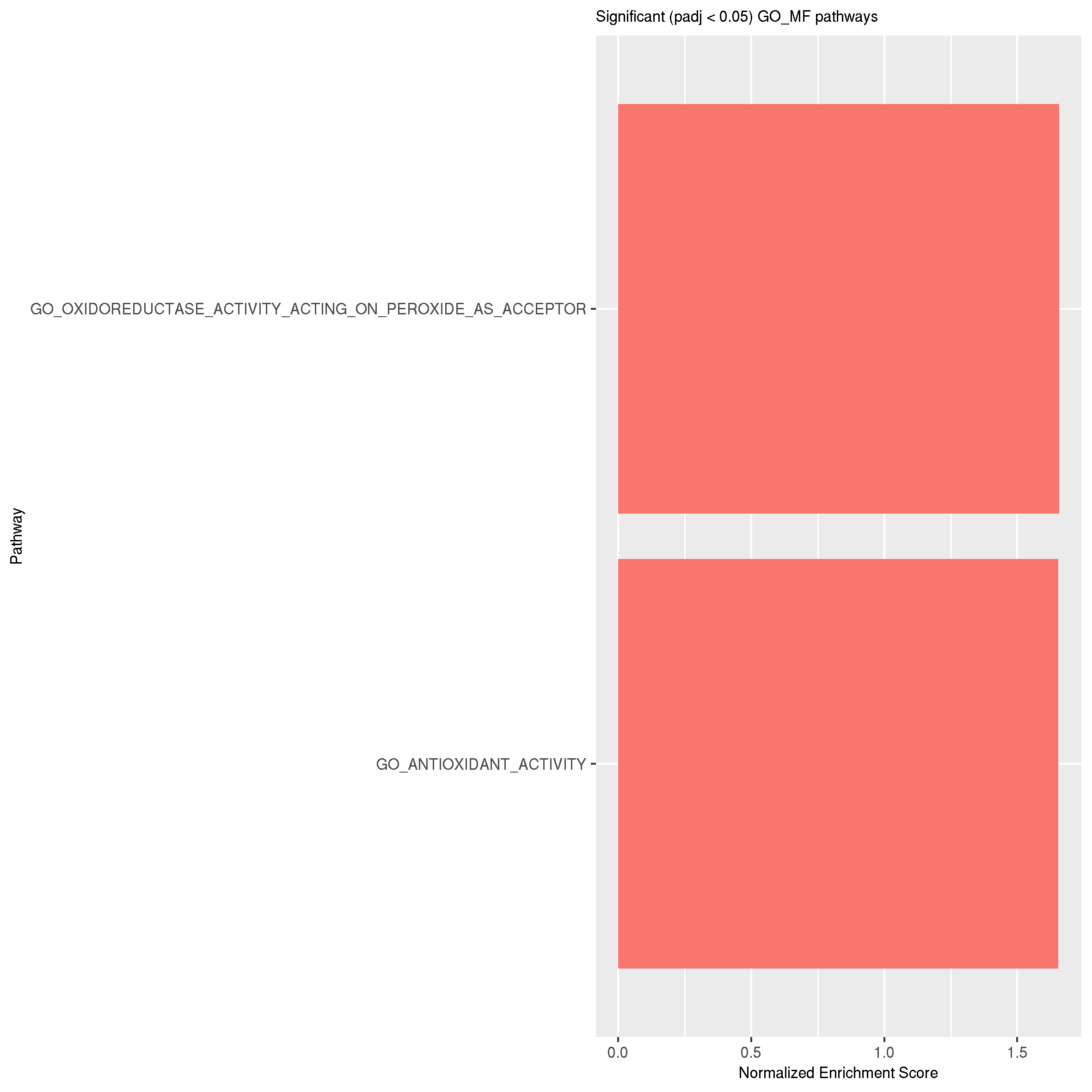


**REFERENCES**

1. Rivas MA, Graham D, Sulem P, Stevens C, Desch AN, Goyette P, et al. A protein-truncating R179X variant in RNF186 confers protection against ulcerative colitis. Nat Commun. 2016 Aug 9;7:12342.

2. Kurki MI, Saarentaus E, Pietiläinen O, Gormley P, Lal D, Kerminen S, et al. Contribution of rare and common variants to intellectual disability in a sub-isolate of Northern Finland. Nat Commun. 2019 Jan 24;10(1):410.

3. McLaren W, Gil L, Hunt SE, Riat HS, Ritchie GRS, Thormann A, et al. The Ensembl Variant Effect Predictor. Genome Biol. 2016 Dec;17(1):122.

4. Karczewski KJ, Francioli LC, Tiao G, Cummings BB, Alföldi J, Wang Q, et al. The mutational constraint spectrum quantified from variation in 141,456 humans. Nature. 2020 May;581(7809):434–43.

5. Vartiainen E, Laatikainen T, Peltonen M, Juolevi A, Mannisto S, Sundvall J, et al. Thirty-five-year trends in cardiovascular risk factors in Finland. International Journal of Epidemiology. 2010 Apr 1;39(2):504–18.

6. Trujillano D, Bertoli-Avella AM, Kumar Kandaswamy K, Weiss ME, Köster J, Marais A, et al. Clinical exome sequencing: results from 2819 samples reflecting 1000 families. Eur J Hum Genet. 2017;25(2):176–82.

7. Karczewski KJ, Weisburd B, Thomas B, Solomonson M, Ruderfer DM, Kavanagh D, et al. The ExAC browser: displaying reference data information from over 60 000 exomes. Nucleic Acids Res. 2017 04;45(D1):D840–5.

8. Stenson PD, Mort M, Ball EV, Evans K, Hayden M, Heywood S, et al. The Human Gene Mutation Database: towards a comprehensive repository of inherited mutation data for medical research, genetic diagnosis and next-generation sequencing studies. Hum Genet. 2017;136(6):665–77.

9. Trujillano D, Oprea G-E, Schmitz Y, Bertoli-Avella AM, Abou Jamra R, Rolfs A. A comprehensive global genotype-phenotype database for rare diseases. Mol Genet Genomic Med. 2017 Jan;5(1):66–75.

10. den Dunnen JT, Dalgleish R, Maglott DR, Hart RK, Greenblatt MS, McGowan-Jordan J, et al. HGVS Recommendations for the Description of Sequence Variants: 2016 Update. Hum Mutat. 2016;37(6):564–9.

11. Richards S, Aziz N, Bale S, Bick D, Das S, Gastier-Foster J, et al. Standards and guidelines for the interpretation of sequence variants: a joint consensus recommendation of the American College of Medical Genetics and Genomics and the Association for Molecular Pathology. Genet Med. 2015 May;17(5):405–24.

12. Kumar A, Kankainen M, Parsons A, Kallioniemi O, Mattila P, Heckman CA. The impact of RNA sequence library construction protocols on transcriptomic profiling of leukemia. BMC Genomics. 2017 Aug 17;18(1):629.

13. Robinson MD, McCarthy DJ, Smyth GK. edgeR: a Bioconductor package for differential expression analysis of digital gene expression data. Bioinformatics. 2010 Jan 1;26(1):139–40.

14. Frazee AC, Pertea G, Jaffe AE, Langmead B, Salzberg SL, Leek JT. Ballgown bridges the gap between transcriptome assembly and expression analysis. Nat Biotechnol. 2015 Mar;33(3):243–6.

15. Pertea M, Pertea GM, Antonescu CM, Chang T-C, Mendell JT, Salzberg SL. StringTie enables improved reconstruction of a transcriptome from RNA-seq reads. Nat Biotechnol. 2015 Mar;33(3):290–5.

16. Liberzon A, Subramanian A, Pinchback R, Thorvaldsdóttir H, Tamayo P, Mesirov JP. Molecular signatures database (MSigDB) 3.0. Bioinformatics. 2011 Jun 15;27(12):1739–40.

17. The Gene Ontology Consortium. The Gene Ontology Resource: 20 years and still GOing strong. Nucleic Acids Res. 2019 Jan 8;47(D1):D330–8.

18. Kanehisa M, Sato Y, Kawashima M, Furumichi M, Tanabe M. KEGG as a reference resource for gene and protein annotation. Nucleic Acids Res. 2016 Jan 4;44(D1):D457-462.

19. Jassal B, Matthews L, Viteri G, Gong C, Lorente P, Fabregat A, et al. The reactome pathway knowledgebase. Nucleic Acids Res. 2020 Jan 8;48(D1):D498–503.

20. Zhang Y, Topham DJ, Thakar J, Qiu X. FUNNEL-GSEA: FUNctioNal ELastic-net regression in time-course gene set enrichment analysis. Bioinformatics. 2017 Jul 1;33(13):1944–52.

21. Rodriguez JM, Rodriguez-Rivas J, Di Domenico T, Vázquez J, Valencia A, Tress ML. APPRIS 2017: principal isoforms for multiple gene sets. Nucleic Acids Research. 2018 Jan 4;46(D1):D213–7.

22. Lindeboom RGH, Vermeulen M, Lehner B, Supek F. The impact of nonsense-mediated mRNA decay on genetic disease, gene editing and cancer immunotherapy. Nat Genet. 2019 Nov;51(11):1645–51.

23. Howe KL, Achuthan P, Allen J, Allen J, Alvarez-Jarreta J, Amode MR, et al. Ensembl 2021. Nucleic Acids Research. 2021 Jan 8;49(D1):D884–91.

24. Auwera G van der, O’Connor BD. Genomics in the cloud: using Docker, GATK, and WDL in Terra. 2020.

25. Gat Y, Schuller JM, Lingaraju M, Weyher E, Bonneau F, Strauss M, et al. InsP6 binding to PIKK kinases revealed by the cryo-EM structure of an SMG1-SMG8-SMG9 complex. Nat Struct Mol Biol. 2019 Dec;26(12):1089–93.

26. Waterhouse A, Bertoni M, Bienert S, Studer G, Tauriello G, Gumienny R, et al. SWISS-MODEL: homology modelling of protein structures and complexes. Nucleic Acids Res. 2018 Jul 2;46(W1):W296–303.

27. Teran NA, Nachun DC, Eulalio T, Ferraro NM, Smail C, Rivas MA, et al. Nonsense-mediated decay is highly stable across individuals and tissues. Am J Hum Genet. 2021 Jun 29;S0002-9297(21)00232-9.
